# spatialstein: An Open-Source Workflow for Annotation, Deconvolution, and Spatially Aware Segmentation of Mass Spectrometry Imaging Data

**DOI:** 10.1021/acs.analchem.5c04737

**Published:** 2026-01-02

**Authors:** Michał Aleksander Ciach, Dan Guo, Kylie Ariel Bemis, Dirk Valkenborg, Olga Vitek, Anna Gambin

**Affiliations:** † Department of Applied Biomedical Science, Faculty of Health Sciences, 269001 University of Malta, Msida MSD 2080, Malta; ‡ Faculty of Mathematics, Informatics and Mechanics, 49605 University of Warsaw, Warsaw 02-097, Poland; § Data Science Institute, 54496 Hasselt University, Diepenbeek 3590, Belgium; ∥ Khoury College of Computer Sciences and Barnett Institute, 1848 Northeastern University, Boston, Massachusetts 02115, United States

## Abstract

Mass Spectrometry Imaging (MSI) data sets are markedly different from optical images. However, analysis algorithms often overlook the intricacies of this kind of data. In MSI, a frequently observed phenomenon is variability in signal intensity between pixels caused by factors other than differences in analyte concentrations. Another common issue is the presence of ions with overlapping isotopic envelopes resulting in isobaric interference. The first factor causes random variations of the signal from the same anatomical regions. The second can cause the spatial distribution of a single peak to represent a mixture of spatial distributions of several analytes. Both factors affect the accuracy of data analysis methods such as MSI segmentation. In this article, we demonstrate that accounting for the intricate structure of MSI data can increase the accuracy of the analysis results. We propose an approach that leverages recent advancements in computational mass spectrometry to separate overlapping isotopic envelopes and mitigate pixel-to-pixel variability of signal intensity. We implemented the approach in spatialstein, an open-source workflow that provides a tentative annotation of an MSI data set with molecular formulas, generates a deconvolved ion image for each annotated ion, and segments each deconvolved ion image into regions of distinct intensity of the corresponding analyte. The structure of the workflow is modular, making it highly modifiable and applicable, whole or in parts, to other studies. The spatialstein workflow is available at https://github.com/mciach/spatialstein.

## Graphical abstract

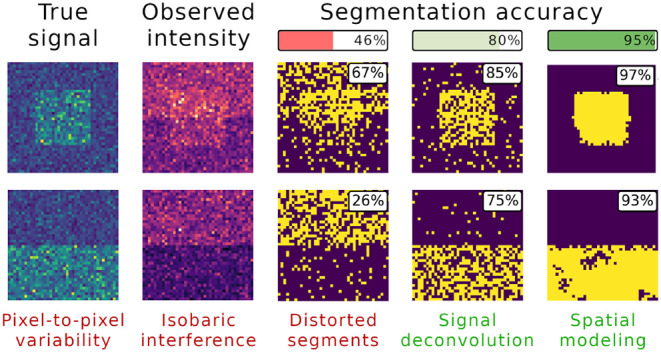

## 1 Introduction

Harnessing the size and complexity of Mass Spectrometry Imaging (MSI) data requires the use of dedicated algorithms and data analysis methods.^1–3^ Among the most popular methods is *segmentation*, i.e., identification of regions with characteristic chemical compositions.^1,2,4^ Such regions are usually interpreted as corresponding to biologically or anatomically distinct parts of the sample, such as tissues, lesions, tumors, etc.^5,6^ This approach offers a simple and reliable approach to distinguish and characterize anatomical regions and identify novel biomarkers.^7–9^

However, the correspondence between segments and anatomical regions can be hindered by a number of phenomena inherent to mass spectrometry and MS imaging technology. A particular phenomenon typical for MSI data is referred to as *pixel-to-pixel variability* of signal intensity.^10,11^ The intensities of signals in MSI data can be influenced by multiple factors, such as the number of ionized molecules, the number of ions transferred to and through the spectrometer, ionization suppression and/or matrix inhomogeneity.^12^ Since these are different for every pixel and cannot be fully controlled, there is a variation of the signal intensity between pixels that is caused by other factors than the differences in chemical composition. Pixel-to-pixel variability of the intensity^10,11^ is a recognized factor that decreases the accuracy and usability of MSI data segmentation.^4,13^

The second characteristic feature of MSI data is the presence of *overlapping isotopic envelopes* (OIE) of ions with similar masses. The isotopic peaks of such envelopes can merge, especially if the resolving power is limited. When a single peak corresponds to several analytes with different spatial distributions, the resulting segmentation combines several regions with different chemical compositions, decreasing its correctness and usability. The problem of OIEs has been extensively studied in many areas of mass spectrometry,^14–20^ both from the experimental and computational perspectives. In the context of MSI, it has been attracting a growing attention with the development of data analysis approaches such as ratio images,^21^ charge-deconvolution algorithms for lower resolution data,^22^ approaches to resolve isobaric interferences using spatial information,^23^ and experimental advancements such as Tandem MSI and Ion Mobility MSI. However, further research is needed to better understand the potential impact of OIEs on downstream analyses such as data segmentation and to develop improved computational methods to mitigate it.

In this manuscript, we present spatialstein, a workflow for segmentation of MSI data that accounts for OIEs and pixel-to-pixel variability (Figure 1). We show that methods originally developed for optical images that do not explicitly model these phenomena can result in highly inaccurate segmentation. In particular, they can return segments that do not correspond to any anatomical region or, in extreme cases, suggest high concentration specifically outside of the true localization region of an analyte. On the other hand, separating OIEs and mitigating the pixel-to-pixel variability allows for a more accurate segmentation with an improved correspondence to the underlying anatomical regions. Additionally, spatialstein provides a tentative assignment of molecular formulas of ions detected in the analyzed MSI data set, as well as the deconvolved ion images of these ions.

**Figure 1:**
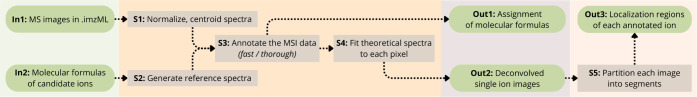
The spatialstein workflow takes as input a list of MSI data sets (step In1) and a set of candidate molecular formulas of ions (step In2), and outputs a tentative assignment of molecular formula to peaks (Out 1), a set of deconvolved single ion images (Out 2), and the regions of distinct average intensity in each image (Out 3). The workflow starts with preprocessing the MSI data sets (step S1) and generating theoretical spectra of the candidate ions (step S2). Next, the MSI data sets are annotated using masserstein to detect which candidate ions are present and provide a tentative assignment of molecular formulas (step S3). The isotopic envelopes of the annotated ions are then deconvolved with masserstein (step S4) for a robust estimation of their signal. These steps result in a list of deconvolved ion images for each annotated ion (step Out1) and a list of corresponding molecular formulas (step Out2). In a downstream analysis, the estimated signals are segmented using a spatially aware algorithm spatialDGMM (step S5) to find distinct regions of analyte concentration in the MSI data (step Out3).

## 2 Materials and Methods

### 2.1 The masserstein Algorithm

A common approach to estimate the proportions of ions with OIEs is to obtain their reference spectra (e.g., by theoretical prediction) and fit them to the data in a way that the coefficients of the fitted combination are equal to the estimated proportions of signal corresponding to each ion. This approach is referred to by several different names in various fields of spectroscopy, including linear deconvolution, linear decomposition, curve fitting, or linear regression of spectra.^14,24,25^ Since linear deconvolution uses the entire isotopic envelopes rather than single peaks, it can resolve overlapping signals and provide a better estimation of the relative proportions.

One of the tools for linear deconvolution of spectra is the Python 3 package masserstein.^24^ It is a general-purpose tool suitable for various types of spectroscopic data, including mass and NMR spectra of various types of molecules.^25,26^ The advantage of this tool over the competing approaches is the use of optimal transport theory for matching experimental and theoretical signals. This makes it robust to differences in resolution of the compared spectra, line shape distortions, small calibration errors and shifts in peak locations caused e.g., by centroiding inaccuracies.^26–29^ Solutions based on optimal transport theory were shown to outperform competing approaches in terms of the accuracy of the estimated proportions of mixture consituents, both in NMR spectroscopy^26^ and mass spectrometry.^30^

Mass shifts, centroiding inaccuracies, and different resolutions of compared spectra complicate typical workflows for MSI data analysis. This is because many analysis methods are sensitive to small differences in the *m*/*z* values of compared peaks. A common way to solve these problems is to bin mass spectra and align them between pixels. A particular advantage of masserstein in this context is that, thanks to the aforementioned robustness, it does not require these operations. Processing spectra without the need for mass binning and alignment avoids the loss of information in the preprocessing stage and simplifies the overall workflow. This makes masserstein a natural candidate for a linear deconvolution tool for MSI data. The downside to the optimal transport paradigm is an increased computational time, which can be mitigated by an appropriate annotation of signals to decrease the number of features in the mass spectra.

The input to masserstein is a mass spectrum in either profile or centroid mode and either a library of reference spectra or a list of chemical formulas of the analytes of interest. In the latter case, theoretical reference mass spectra are automatically calculated with IsoSpec.^31^ The output is a list of estimated proportions of the ions, as well as the remaining signal not matched to any of the reference spectra. Ion signals estimated with masserstein can directly replace monoisotopic peak intensities as input to most downstream analysis methods such as image segmentation.

The software requires the user to specify two parameters that depend on the mass accuracy, resolving power of the instrument, and the intensity limit of detection. The parameter *k*_mixture_ is the penalty for removing excess signal in the experimental spectrum. The parameter *k*_components_ is the penalty for removing excess signal in the theoretical spectra. The values of both parameters are expressed as the expected difference in *m*/*z* between corresponding theoretical and experimental signals:^25,26^ *k*_mixture_ is the expected maximum *m*/*z* window around an experimental peak to match it to a signal from any theoretical spectrum, and *k*_components_ is the expected maximum *m*/*z* window around a theoretical peak to match it to an experimental one. However, for any values of the κ parameters, masserstein can further adjust the distances between matched signals based on the shapes of the isotopic envelopes.^25^ This makes the κ parameters flexible thresholds rather than strict *m*/*z* windows commonly used in other approaches, which further increases the robustness of the approach and allows for a more flexible tuning of the parameters.^30^

### 2.2 The Cardinal Package and spatialDGMM Segmentation Algorithm

One of the most common approaches to MSI data analysis is segmentation, which partitions data into regions of distinct chemical composition.^4,5,32^ MSI data segmentation methods can be roughly divided into two types: multivariate and univariate. Multivariate segmentation methods, such as spatial shrunken centroids^4^ implemented in the open-source Cardinal package for MSI data analysis,^33^ partition the data set based on multiple features, such as peaks. In contrast, univariate segmentation methods consider one feature at a time.

The combination of multiple features in multivariate methods can reinforce the signal in the spectra, thereby providing a more accurate segmentation. However, multivariate methods rely on the assumption that the spatial distributions of all the analytes can be partitioned into a single common set of segments. This assumption is often unrealistic, because MSI data sets usually contain ions with distinct, potentially overlapping regions of localization. Although multivariate approaches provide a comprehensive characterization of molecular differences in a tissue, univariate segmentation can be more appropriate when the goal is to localize and quantify specific biomarkers or metabolites with high precision. It allows for clearer biological interpretation, easier comparison between tissue regions, and reduces the risk of overfitting or misleading correlations among ions. Therefore, univariate methods may provide more meaningful segmentations for each ion, which can be later summarized using methods such as hierarchical clustering.^13,34^ The drawback of univariate segmentation methods is that they are more prone to artifacts of OIEs and to pixel-to-pixel variability of the signal.

The sensitivity of segmentation methods to pixel-to-pixel variability has already been noted and addressed by some authors, who have demonstrated that accounting for spatial relations between pixels can mitigate this effect and improve the quality of segmentation.^4,10,13,35,36^ Accordingly, several algorithms for spatially aware segmentation methods were developed. However, most of the efforts focused on multivariate segmentation. The spatialDGMM algorithm, implemented in the Cardinal package,^33^ addressed the lack of spatially aware univariate segmentation methods. The algorithm is based on Bayesian approach to Gaussian Mixture Models.^13^ The use of Gaussian Mixture Models makes it suitable for segments of different sizes, and the Bayesian approach allows for applying a spatial filter to posterior probabilities of segment assignment in order to mitigate the effect of pixel-to-pixel variability.

### 2.3 The spatialtein Workflow

In this work, we combine masserstein and spatialDGMM into spatialstein, a workflow for linear deconvolution and spatially aware segmentation of MSI data robust to OIE and pixel-to-pixel variability. The overview of the proposed workflow is shown in Figure 1. The input to the workflow is an MSI dataset in .imzML format, a list of chemical formulas of the analytes of interest, and their ionization adducts. The output of the workflow is a set of molecular formulas of detected analytes, the corresponding deconvolved ion images, and the segmentation results of each image.

The analysis proceeds in four main steps:

(1) A simple preprocessing, including normalization and centroiding (but without the need for *m*/*z* binning or alignment thanks to the use of masserstein)
(2) Tentative data annotation (peak assignment), either using a “fast” strategy based on the average spectrum or a “thorough” strategy based on annotating each pixel separately and pooling annotations
(3) Signal quantification through a linear deconvolution of spectra, using masserstein to obtain an accurate signal estimation in the presence of OIE without the need for *m*/*z* binning and alignment
(4) Image segmentation with a spatially aware algorithm spatialDGMM to mitigate pixel-to-pixel variability of the signal caused, e.g., by random differences in ion statistics

Below, we describe each step of the workflow in detail, including example data sets used for its evaluation and how to find appropriate values of parameters at each step. A further discussion of selected steps, as well as possible alternative approaches and their advantages and disadvantages, is available in the Supporting Information. There, we also discuss some additional considerations and common pitfalls that can lead to incorrect results, including model violations (i.e., discrepancies between the theoretically predicted and experimentally measured signals) and how to identify and correct them.

All the computations were performed on a personal laptop computer with an 11th Gen Intel Core i7–11850H @ 2.50 GHz processor and 16 GB RAM.

### 2.4 Input Data Sets (Figure 1, Step In1)

To evaluate the performance of spatialstein, we have used it to analyze three MSI data sets. The first data set, referred to as the *mouse cerebellum*, was downloaded from the MetaboLights dabatase^37^ (ID MTBLS487). It is a relatively small MSI data set (81 × 21 pixels, 1701 pixels in total) of a tissue section of a mouse cerebellum, obtained on a MALDI Orbitrap instrument with a pixel size of 50 μm, mass resolving power R = 60 000 at 200 *m*/*z*, and low amount of contaminants and background noise.^38^ The data set contains four well-delineated anatomical regions with distinct lipid concentrations that are relatively easy to find by segmentation: the background, the white matter, the granular layer and the molecular layer; tissues were determined with histological staining in the original work^38^ (Figure 2A).

**Figure 2:**
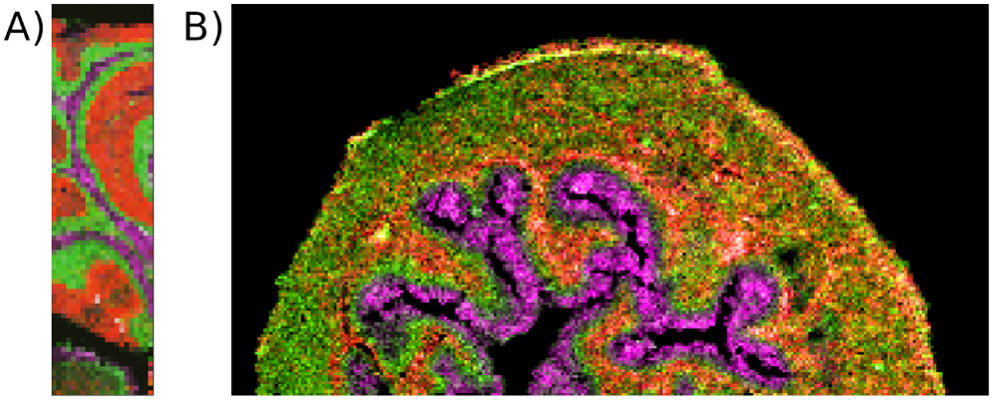
The mouse cerebellum and mouse bladder data sets used to evaluate spatialsteinin practical applications. A) An overlay of three single ion images from the mouse cerebellum MSI data set: 744.49 Da, concentrated in the white matter (magenta); 820.53 Da, concentrated in the granular layer (green); and 856.58 Da, concentrated in the molecular layer (red). B) An overlay of three single ion images from the mouse bladder MSI data set: 842.55 Da, concentrated in the urothelium (magenta); 851.64 Da, concentrated in the lamina layer surrounding the urothelium (red); and 422.93 Da, concentrated in the myofibroblast layer between the lamina and the urothelium, and in the muscle tissue surrounding the lamina (green).

The second data set, referred to as the *mouse bladder*, was downloaded from the PRIDE database^39^ (ID PXD001283). It is a larger data set (260 × 134 pixels, 34840 pixels in total) obtained from a sample of a mouse bladder^40^ on a MALDI LTQ Orbitrap instrument with a pixel size of 10 μm, mass resolving power of R = 30 000 at 400 *m*/*z*, a noticeable presence of matrix adducts, and overall more complex spectra than the mouse cerebellum data set (see the average spectra in Figure S1). This is an example of a more challenging input for segmentation. It contains 7 main regions: the background, the adventitial layer, the muscle tissue, the lamina propria, the myofibroblast layer, the urothelium, and the umbrella cells; tissues were determined with histological staining in the original work^40^ (Figure 2B).

The third data set, referred to as the *simulated data set*, was prepared as a part of this work to illustrate potential pitfalls associated with MSI data segmentation. It is a relatively small data set (40 × 40 pixels, 1600 pixels in total) with three lipid ions with potassium adducts: PC(38:1) (C_46_H_90_NO_8_PK; 854.603 Da), PA(44:0) (C_47_H_93_O_8_PK; 855.624 Da), and PC(38:0) (C_46_H_92_NO_8_PK; 856.619 Da). The first lipid was assumed to be concentrated in the top half of the sample; the second in the bottom half; and the third in a 20 × 20 center square, resulting in four distinct regions (see Table 1 and Figure 3). This is a particularly challenging data set that presents many opportunities for segmentation methods to produce inaccurate results.

**Table 1:** Average Numbers of Lipid Ions in Regions of the Simulated MSI Data Set

	PC (38:1)	PA (44:0)	PC (38:0)
Region 1	10 000	2 000	1 000
Region 2	1 000	4 000	1 000
Region 3	10 000	2 000	2 000
Region 4	1 000	4 000	2 000

**Figure 3:**
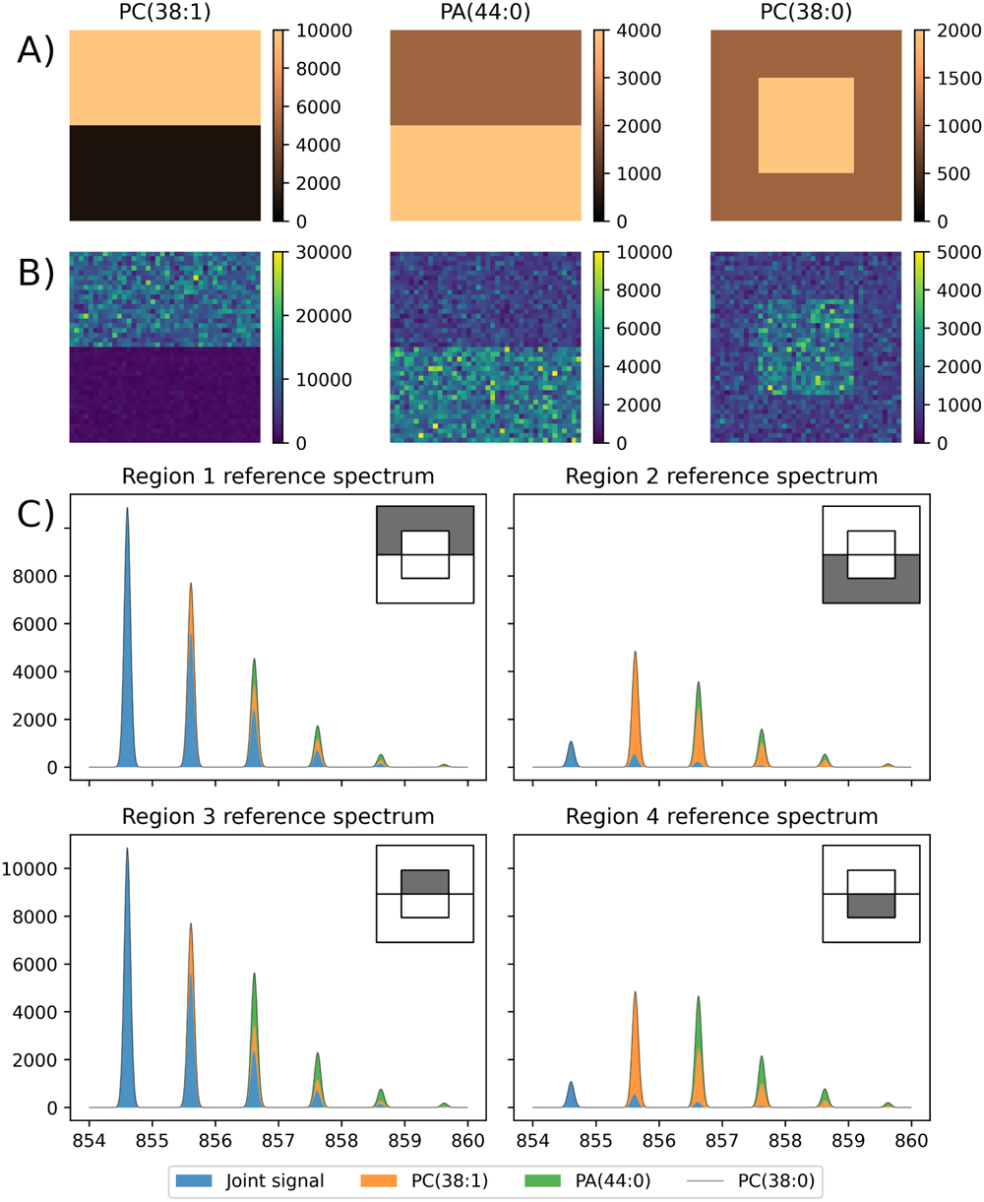
The simulated data set used to evaluate the performance of spatialsteinin particularly challenging scenarios. (A) The reference images of lipid concentrations used to generate the simulated MSI data set; color intensity represents the average number of ions. B) The distribution of the simulated number of ions of each lipid in the simulated MSI data set, showing pixel-to-pixel variability. C) The average spectra of each distinct region of the data set, showing OIEs. The corresponding region of the MSI data set is highlighted in the top-right corner of each spectrum.

To prepare the simulated data set, a reference optical image was drawn manually in the GNU Image Manipulation Program (Figure 3A). Next, for each pixel, the number of ions of each lipid species was drawn from a Negative Binomial distribution with the average value depending on the region (listed in Table 1) and a coefficient of variation of 20% (Figure 3B). The reference isotopic envelopes of the three lipids were generated with masserstein using IsoSpec.^31^ Next, we simulated how ion statistics influence the shape of the isotopic envelopes. For each lipid in each pixel, the measured intensity of isotopic peaks was simulated by drawing samples from a multinomial distribution, with the numbers of trials equal to the drawn numbers of lipid ions in each pixel, and probability vectors proportional to the peak heights of the reference isotopic envelope. After simulating the intensity, the isotopic envelopes of the three lipids were added together (separately in each pixel). To simulate additional signals coming from other ions or the background noise, to each spectrum individually we added 10 randomly located peaks, jointly accounting for 10% of the intensity of the spectrum. A Gaussian filter was then applied to the pixel spectra to simulate a limited resolving power equal approximately 7000 at *m*/*z* 800 (fwhm = 0.12 Da) (Figure 3C). The code to reproduce the simulation is available on the spatialstein website https://github.com/mciach/spatialstein.

### 2.5 Libraries of Reference Spectra (Figure 1, Steps In2 and S2)

The spatialstein workflow can be used with either a library of reference spectra or a library of molecular formulas of interest. In the latter case, reference spectra are calculated using IsoSpec.^31^ Since IsoSpec can calculate the theoretic al isotopic envelope of any molecular formula, the user can supply any library of formulas of interest to spatialstein. Consequently, spatialstein is capable of analyzing ions with different adducts, as well as negatively charged ions in MSI data sets collected in negative mode. Furthermore, spatialstein can be used to analyze molecules other than lipids, e.g., peptides or polymers, thanks to the use of general-use software components that have been validated in multiple research settings.^13,30^ However, we note that the library of candidate ions for annotation must strike a balance (see also the discussion in ref. 30). Insufficiently large libraries may lead to false-negative results (failure to detect an ion that is present in the data set), while excessively large libraries may lead to false-positive results (spurious detection of ions which are absent from the spectra, caused, e.g., by fitting to the background noise or fitting multiple ions to a single isotopic envelope).

In this study, to show an example application of spatialstein, we created a relatively small library of glycerophospholipid and sphingolipid ions with potassium adducts, because such ions were reported in the original studies in which the mouse bladder and cerebellum data sets were published. Furthermore, isobaric interference due to OIEs is a recognized problem for these molecules^18,41^ allowing us to test the performance of spatialstein.

Molecular formulas of glycerophospholipids and sphingolipids were downloaded from the LIPID MAPS database^42^ (accessed March 28, 2022; 3571 unique formulas corresponding to 14504 lipid IDs within 30 lipid subclasses). Formulas containing elements other than CHNOP were discarded, leaving 3523 formulas corresponding to 14454 lipid IDs. The remaining formulas were used to calculate theoretical spectra of lipid ions with potassium adducts using IsoSpec^31^ in masserstein (peak intensity threshold = 0.05). The spatialstein workflow allows the user to restrict the analyzed *m*/*z* range in order to focus on a selected group of molecules and speed up the computations. To test this functionality, spectra with the monoisotopic mass lower than 700 Da or greater than 900 Da were discarded, resulting in 1206 theoretical spectra, corresponding to 6922 different lipid IDs within 22 subclasses. We refer to the remaining 1206 ions as the *candidate ions*.

For the simulated data set, the reference library consisted of the three lipid ions used in the simulation.

### 2.6 Data Preprocessing (Figure 1, Step S1)

For the mouse bladder and cerebellum data sets, all pixel spectra were normalized by equalizing their total ion current in order to account for artifacts such as varying laser intensity, ionization efficiency or matrix inhomogeneity.^43^ The total ion current was calculated by numerical integration of intensities in profile mode (trapezoid method). Next, all pixel spectra were restricted to the mass range of 700 to 900 Da. Since the simulated data set did not include artifacts causing differences in total ion current of the pixel spectra, it was not normalized.

The pixel spectra were centroided using the masserstein package according to a tutorial from the project’s website. For the mouse cerebellum and bladder data sets, the peaks were integrated within the full width at half-maximum of signal intensities, with a maximum width of the integration region 0.2 Da. For the simulated data set, the peaks were integrated within the full width at 20% of maximum intensity, maximum integration region 0.4 Da. The correctness of the results was assessed by a visual inspection of randomly selected pixel spectra overlaid in both profile and centroid modes, as well as by comparing single ion images of selected lipid ions generated from MSI data sets in centroided and profile mode (Supplementary Figure S2).

### 2.7 Peak Assignment (Figure 1, Steps S3, Out1)

As a part of this work, we have designed, implemented and evaluated two annotation strategies that differ by their sensitivity and computational complexity. The first one, which we call “*fast*” (or, equivalently, *average, then annotate*), resembles the approach for MSI data annotation based on accurate mass matching. For each data set, theoretical spectra from the library were fitted to the centroided average spectrum of the data set using masserstein. Stringent mass accuracy criteria were used in order to select only the best-fitting ions (*k*_mixture_ = 0.005, *k*_components_ = 0.01). With these values of the κ parameters, the proportions of analyte abundances estimated by masserstein may not be accurate enough for quantification (because the threshold of 0.005 Da can be too stringent to capture all the signal from an analyte), but can be used as a rough indication of the presence or absence of an analyte. Accordingly, a formula was assigned if it had a nonzero estimated proportion. This strategy resulted in 31 and 128 formulas, respectively, for the bladder and cerebellum data set, and the computations took 1 and 5 s.

The second strategy, which we call “*thorough*” (or, equivalently, *annotate, then average*), was designed to detect more ions that could be hidden within overlapping isotopic envelopes in the average spectra or present in low amounts in the data, at the cost of an increased computational time. An advantage of this strategy is the use of spatial information to refine the annotation. First, the reference spectra from the library were fitted to each pixel spectrum separately using masserstein with the same stringent mass accuracy criteria. The computations took 92 s and 10 min on 16 CPUs for the cerebellum and the bladder, respectively. Next, in each data set, the estimated lipid ion proportions were averaged over all pixels to calculate the average proportion of the ion in the data set. This average mimics the estimation of proportions from an average spectrum: while in the “fast” strategy we first take the average of all spectra and then estimate the proportions of analytes, in the “thorough” strategy we first estimate the proportions of analytes in each spectrum and then average out the proportions. Since this strategy is sensitive to random noise, annotating all ions with nonzero proportions (205 and 236 for the bladder and cerebellum, respectively) was not feasible. A histogram of averaged estimated proportions showed a clear bimodal distribution, indicating a natural threshold of 10^–9^ for both data sets (Supplementary Figure S8). Furthermore, ions with proportions under this threshold were detected in low numbers of pixels (Supplementary Figure S3). Therefore, we assigned ions with estimated average proportions greater than 10^–9^, which produced 180 and 209 annotated ions for the bladder and cerebellum, respectively. A comparison of the two strategies is discussed further in the Supporting Information. The subsequent analyses in this manuscript use the results from the *annotate, then average* strategy.

In the mouse bladder data set, both approaches correctly identified ions SM(34:1), PC(32:0), PC(34:1) and PC(38:4) that were experimentally annotated in the original work,^40^ corroborating a correct annotation. However, we note that the other annotations obtained using purely computational methods on MS1 spectra and not confirmed by tandem MS may contain false positives and false negatives and should not be viewed as definitive assignments. Therefore, we refer to these annotations as *tentative*.

The annotation step was skipped for the simulated data set, and the three lipids used to generate it were used in the subsequent steps. This was done to ensure that the results obtained on the simulated data set evaluated only the effects of OIE and pixel-to-pixel variability.

### 2.8 Estimation of Lipid Ion Proportions (Figure 1, Steps S4, Out2)

For the mouse bladder and cerebellum data sets, the appropriate values for the masserstein parameters *k*_mixture_ and *k*_components_ were determined by first identifying three lipids with no observed OIE in the average spectra of either data set: PC(32:0), PC(34:1), and PC(38:4), referred to as the test lipid ions. Due to the lack of OIE interference, appropriate parameter values should result in deconvolved ion images of the three lipids identical to their single-ion images. Accordingly, the optimal values of the parameters were found by comparing the deconvolved and the single-ion images of these lipids and identifying parameter values which give the highest similarity measured by the correlation of the signal.

To limit computational complexity, 1000 pixels were randomly sampled. For each combination of κ values in the range of 0.002, 0.004, 0.006, ..., 0.03, the reference isotopic envelopes of the three lipids above were fitted to the spectra of the sampled pixels. Next, the correlations were calculated between the intensities of the monoisotopic peaks and the proportions estimated with masserstein. In agreement with the results for other applications of masserstein,^30^ a relatively large range of parameter values resulted in accurate estimation results (Supplementary Figure S4). The parameters *k*_mixture_ = 0.012, *k*_components_ = 0.016, which resulted in correlation values of 0.99, 0.98, and 0.97 for the bladder and 0.99, 0.99, and 0.99 for the cerebellum for the three test lipid ions, respectively, were selected for further processing. The correctness of these parameter values was further verified by a visual comparison of the single-ion and the deconvolved ion images for the three test lipid ions (Supplementary Figure S5).

Using these parameter values, the reference spectra of all ions obtained in the peak assignment step were fitted to each pixel spectrum of the mouse bladder and cerebellum data sets, resulting in deconvolved ion images for the ions (Figure 1, Out2). The computations took 29 s and 301 s for the mouse cerebellum and bladder data set, respectively.

For the simulated data set, we used parameter values *k*_mixture_ = 0.2, *k*_components_ = 0.5. The higher values of the parameters reflect a lower resolution of the spectra in the simulated data set. Since these parameters allowed for a sufficiently accurate deconvolution, we did not optimize them further.

### 2.9 Univariate Segmentation of MSI Data Sets (Figure 1, Steps S5, Out3)

The deconvolved ion images were saved in .imzML format and loaded into the R programming language using the Cardinal v3.6.5 library.^33^ Next, the data sets were segmented using the spatialDGMM function from the same library (Supplementary Figure S5) into low- and high- intensity segments (k = 2) with spatial smoothing to mitigate pixel-to-pixel variability (*r* = 3, β = 6 for the simulated data set; r = 1, β = 4 for the cerebellum data set; r = 3, β = 6 for the bladder data set). The *r* and β parameters were selected based on a visual inspection of the segmentation results and a comparison with the deconvolved ion images for 12 randomly selected lipids in the mouse bladder and cerebellum data sets, and the 3 lipids in the simulated data set.

In order to assess the impact of pixel-to-pixel variability on the segmentation, we have compared the results of spatialDGMM with a simple thresholding of intensity. The K-means algorithm (k = 2) was used to obtain an automatic intensity threshold for each lipid ion separately (treating the intensity of a lipid ion in each pixel as a 1D column vector). Since the K-means algorithm does not use the spatial information about the pixels, we refer to this approach as the spatially naïve segmentation. Accordingly, refer to the spatialDGMM as the *spatially aware* approach.

## 3 Results and Discussion

### 3.1 The Inherent Complexity of MSI Data Complicates the Analysis and Interpretation of Single-Ion Images

To demonstrate the potential pitfalls of the conventional approaches to MSI data segmentation, in particular on lower resolution instruments, we simulated a 40 × 40 pixel MSI data set with mass resolution R = 7000 at *m*/*z* 800, consisting of three lipids: PC (38:1), 854.603 Da; PA(44:0), 855.624 Da; and PC(38:0), 856.619 Da. PC(38:1) was localized in the top half; PA(44:0) in the bottom half; and PC(38:0) in a 20 × 20 square in the middle of the image (Figure 3A,B). The monoisotopic peak of PA (44:0) was within the isotopic envelope of PC(38:1), and the monoisotopic peak of PC(38:0) was within the isotopic envelopes of PC(38:1) and PA(44:0) (Figure 3C).

For PC (38:1), which was not subject to OIE interference, the monoisotopic peak intensity accurately reflected the spatial localization of this lipid. The correlation between the true number of simulated ions of this lipid and its monoisotopic peak intensity was equal to ρ = 0.99 (Figure 4), indicating that the monoisotopic peak intensity accurately reflected the localization of the lipid in the sample.

**Figure 4:**
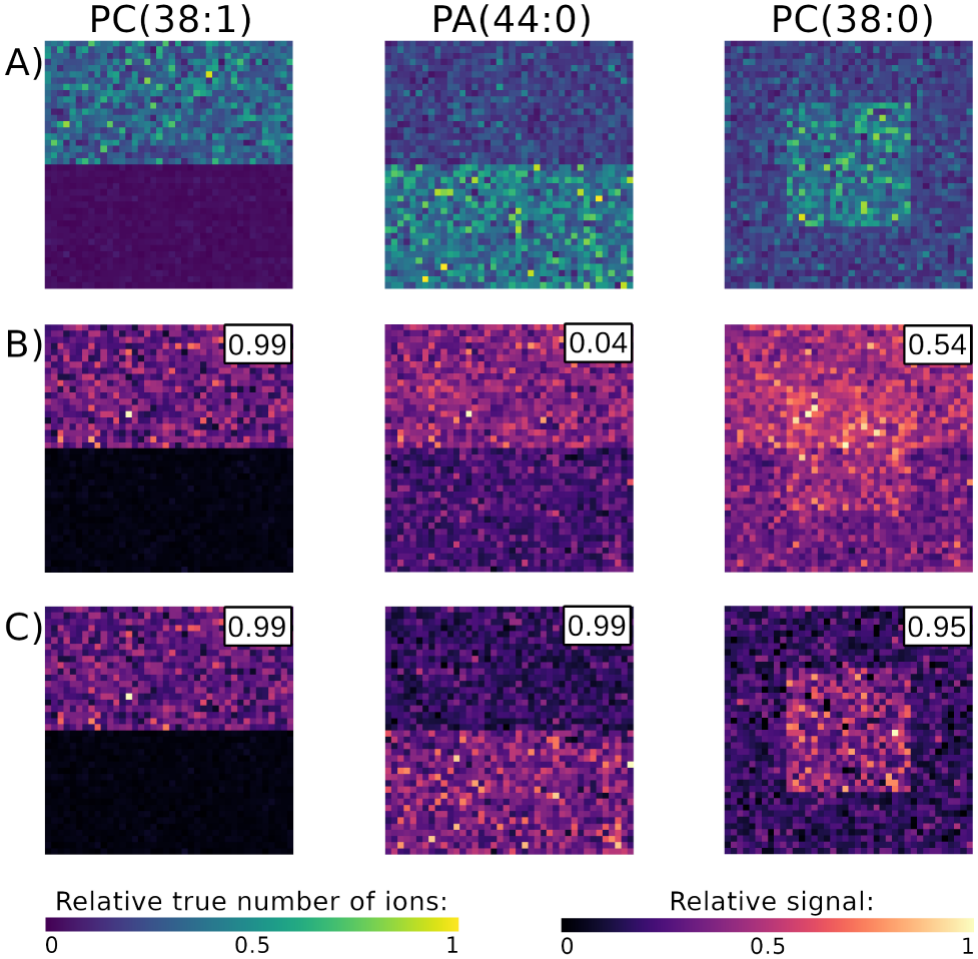
Simulated data set: overlapping isotopic envelopes distorted the apparent spatial distribution of analytes. The correlations between the true number of simulated ions and either the monoisotopic peak area or the signal estimated via linear deconvolution are shown in the top-right corners of the images. A) The true number of simulated ions of each lipid. B) The monoisotopic peak intensity of PA(44:0) was overshadowed by PC(38:1), and that of PC(38:0) was distorted by the two other lipids. C) Separating the overlapping isotopic envelopes with linear deconvolution corrected the spatial distributions.

However, for the two lipids that were subject to the OIE interference, the spatial distributions of their monoisotopic intensities did not reflect their true localizations (Figure 4A,B). The signal of PC(38:0) was spread over the image and did not show any meaningful localization regions. Accordingly, the correlation of the signal and the number of ions was equal to ρ = 0.54. The signal of PA(44:0) was higher in the bottom half of the image, despite the lipid being localized in the top half. This reversal of apparent localization was caused by the high signal intensity of PC(38:1) in the top half, which influenced the apparent intensity of the monoisotopic peak of PA(44:0). Consequently, the correlation between the number of ions and the peak intensity was equal to only ρ = 0.04.

The example demonstrates that OIE interference can introduce extensive changes to the apparent distribution of signal intensity. Influence from high-intensity ions can confound the spatial distribution of the signal, and in extreme cases, overshadow it. In these cases, the signal of analytes needs to be estimated with an approach robust to OIE to avoid incorrect results in downstream analyses.

### 3.2 Linear Deconvolution Identified the Correct Spatial Distributions of Ions in the Presence of OIE

Using spatialstein to separate OIE resulted in an accurate spatial distribution of lipid intensity (Figure 4C). The estimated signal of PA(44:0) was localized in the bottom half and that of PC(38:0) in the center square. The correlation between the true number of simulated ions and the signal estimated with linear deconvolution increased to ρ = 0.99 and 0.94 for PA(44:0) and PC(38:0) respectively. The correlation for PC(38:1) remained unchanged at ρ = 0.99, consistent with the lack of interference due to OIE for this lipid. This demonstrates that linear deconvolution can estimate accurate spatial distributions of analytes even in the presence of severe interferences due to OIE.

To confirm that spatialstein correctly separates overlapping signals in real MSI data sets as well as in simulated ones, we performed a computational experiment in which we temporarily lowered the mass resolutions of the spectra in the mouse bladder data set by applying a Gaussian filter (σ = 0.043 *m*/*z*) to each spectrum in profile mode, effectively broadening the signals. This resulted in merging of the monoisotopic peak of SM(40:1), a lipid located in muscle tissue, with the M+1 peak of PC(36:2), a lipid concentrated in the urothelium (Supplementary Figure S10). The resulting single ion image suggested that SM(40:1) is distributed throughout the whole tissue. Nevertheless, spatialstein was still able to correctly deconvolve the ion image and return the correct spatial distribution of both lipids (Supplementary Figure S10). This result indicates that spatialstein can be a viable alternative to costly high-resolution instruments to resolve overlapping peaks of identified analytes. The remaining results on the mouse bladder data set shown in this work were obtained with the original resolution.

### 3.3 In Experimental Data Sets, Distortions Caused by OIE Were Infrequent but Severe

The annotation step of spatialstein in “thorough” mode discovered 209 tentative lipid ions in the cerebellum data set and 180 in the bladder data set. To validate our results, we have compared them with annotations provided in the METASPACE platform, computed using the pySM algorithm.^44^ At FDR level 10%, METASPACE annotates the mouse cerebellum data set with 49 ions, and the mouse bladder data set with only one ion. To compare these annotations with the results of spatialstein, we selected ions with molecular formulas present in our reference library of candidate ions. At FDR level 10%, this resulted in 21 unique molecular formulas in the mouse cerebellum, all of which were identified by spatialstein in both the fast and the thorough annotation strategies (see Supplementary Figure S6 for a comparison of spatialstein in “fast” and “thorough” mode with METASPACE at 10% and 20% FDR level). No ions from our library were identified in the mouse bladder by METASPACE at FDR level 10%. Furthermore, METASPACE with full LIPID MAPS as a reference library annotates the mouse bladder with only one molecular formula at FDR 10%. The formula corresponds to Apigenin, a plant flavonoid, and likely represents a false positive annotation. This observation supports our view that annotations done on MS^1^ spectra, while useful for preliminary or exploratory analyses, should be regarded as tentative unless confirmed with additional experimental evidence such as Tandem MS. Apigenin was not present in our reference library and, consequently, was not annotated by spatialstein.

Quantifying the signals of the lipid ions with linear deconvolution indicated large differences in the numbers of pixels in which those lipids were present. For the cerebellum data set, the number of pixels in which a given lipid was detected ranged from 1 to 1700, and for the bladder data set, it ranged from 1 to 17836 (Supplementary Figure S7). While lipids present only in a handful of pixels might be biologically interesting, they can also correspond to contaminants, spurious annotations of background noise, or other factors leading to false positive results. Furthermore, the correctness of their estimated spatial distributions is difficult to assess. Therefore, for subsequent analyses, we have selected lipids which were present in at least 400 pixels in the cerebellum and 1000 pixels in the bladder. These numbers of pixels were sufficient to visually identify the anatomical structures in which the lipids localized in the deconvolved ion images. This additional spatial filter resulted in 77 ions in the cerebellum and 44 ions in the bladder. All the 21 ions selected from METASPACE in mouse cerebellum passed the spatial filter.

In both data sets, the signal estimated with the linear deconvolution step of spatialstein was usually highly correlated with the monoisotopic peak intensity, indicating a lack of interference due to OIE (ρ ≥ 0.9) for 57 out of 77 ions in the cerebellum and 28 out of 44 ions in the bladder data set; Supplementary Figure S9). However, in both data sets we also detected lipid ions whose spatial distributions of the monoisotopic peak intensity were different from the spatial distribution estimated with linear deconvolution, indicating interferences from OIE (ρ < 0.8 for 13 ions in the cerebellum and 6 ions in the bladder).

In some cases, interferences due to OIE severely impacted the apparent localizations of lipids. In the mouse cerebellum data set, the single ion image for the peak 783.5716 Da was distributed throughout the whole tissue. However, spatialstein annotated this peak with two lipids: a tentative phosphatidic acid plasmalogen, PA­(O-40:1), C_43_H_85_O_7_P, 783.567 Da, and a tentative sphingomyelin, SM(37:1), C_42_H_85_N_2_O_6_P, 783.578 Da. The spatial distributions of those lipids estimated with spatialstein were complementary, with PA (O-40:1) concentrated specifically in the white matter and SM(37:1) concentrated specifically outside of it (Figure 5). A manual analysis of the isotopic peaks in the profile-mode average spectra of those tissues confirmed the presence of two ions with complementary spatial distributions, corroborating the results of spatialstein (Supplementary Figure S11). Notably, the “fast” annotation strategy (based on the average spectrum) failed to annotate these lipids due to their overlapping isotopic envelopes in the average spectrum.

**Figure 5:**
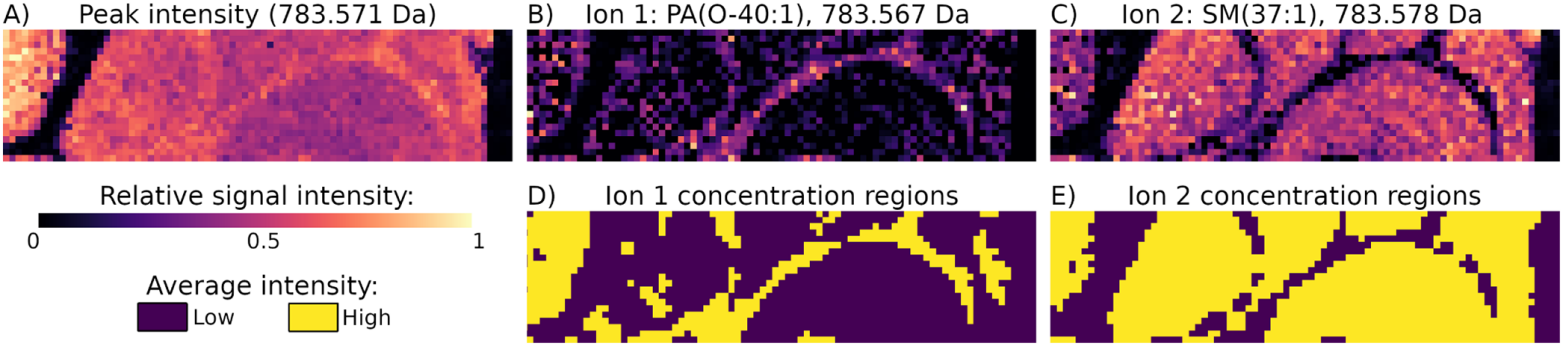
Correcting for the overlapping isotopic envelopes with masserstein and for the pixel-to-pixel variability of intensities with spatialDGMM clearly delineated the concentration regions of lipids in the mouse cerebellum data set. A single peak at 783.571 Da was composed of signals of two ions with complementary spatial distributions: the (tentative) PA­(O-40:1) localized inside the white matter, and the (tentative) SM(37:1) localized outside. A) The single ion image of 783.571 Da, showing a relatively uniform distribution over the tissue; B, C) The deconvolved ion images of two ions contributing to the single ion image at 783.571 Da, showing complementary spatial distributions; D, E) spatialDGMM segmentation of the deconvolved ion images into high- and low-intensity regions.

In the mouse bladder data set, OIE interferences were less severe. Linear deconvolution indicated that some single-ion images showed larger localization regions than true ones. For example, the peak intensity of tentative PC(36:3) (formula identical to PE(39:3)) suggested that the lipid is localized in all of the urothelium tissue. Linear deconvolution indicated that the lipid localizes mostly in the umbrella cells, suggesting that the signal in the remaining part of urothelium may come from other ions (Supplementary Figure S12, cf. Figure 2). After linear deconvolution, the spatial distributions of some lipids were more precise than indicated by their single-ion images. For example, for the tentative PC(35:2) (formula identical to PE(38:2)), localized in the umbrella cells, the single-ion image suggested the presence of the lipid in random individual pixels outside of this tissue. Linear deconvolution, which is more robust to random noise signals, removed the lipid’s signal from these pixels (Supplementary Figure S12).

### 3.4 Pixel-to-Pixel Variability of Signal Intensity Impacted Segmentation in Addition to OIE

To analyze the impact of pixel-to-pixel variability on segmentation, we have segmented the simulated data set using two approaches: a simple intensity thresholding (referred to as the spatially naïve approach) and spatialDGMM segmentation (referred to as the *spatially aware* approach).

After separating OIE, the spatially naïve approach produced a coarse visual identification of the localization regions of the analytes (Figure 6, Supplementary Figure S15). However, this approach did not provide a sufficient level of accuracy for quantitative analyses, as the segments were highly rugged due to pixel-to-pixel variability and contained pixels from different concentration regions. The percentage of pixels correctly assigned to high- and low-concentration segments was 91% for PC (38:1), but only 75% and 85% for PA(44:0) and PC(38:0) respectively (Figure 6, Supplementary Figure S15).

**Figure 6:**
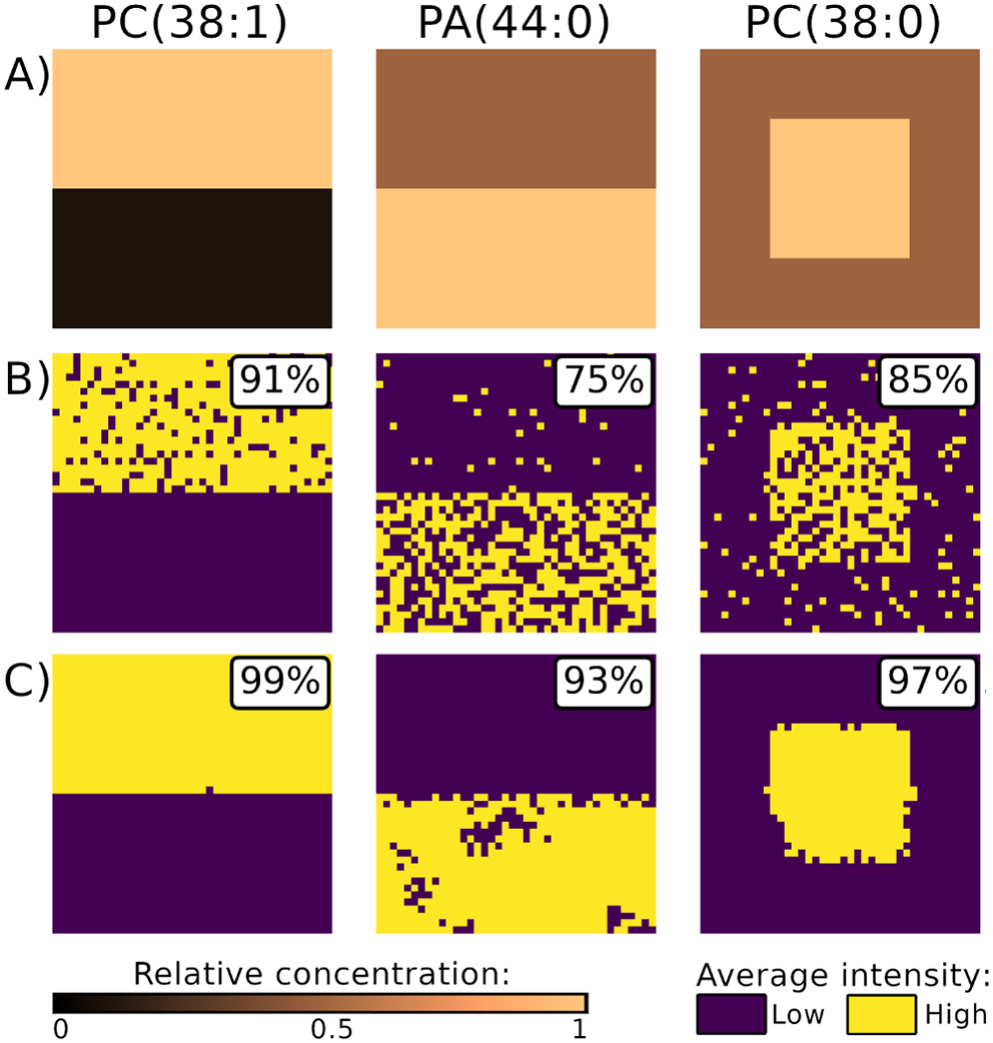
The simulated MSI data set demonstrates how pixel-to-pixel variability of the signal can influence segmentation results even after an accurate estimation of the signal with masserstein. A) The true localization regions of each lipid. B) Due to the pixel-to-pixel variability, segmenting the signals of lipids (estimated with masserstein) by intensity thresholding failed to accurately discover the true concentration regions. The segments were rugged due to random fluctuations of the intensity around the average level. C) The spatialstein workflow discovered the true concentration regions of lipids thanks to linear deconvolution of OIE with masserstein and mitigation of pixel-to-pixel variability with spatialDGMM.

A spatially naïve segmentation of the mouse bladder data set supported the same conclusions. The pixel-to-pixel variability caused highly rugged clusters that allowed only for an approximate visual identification of localization regions (Supplementary Figure S13). For example, in the mouse bladder data set, the tentative PC(32:0) did not have interference due to OIE, as evidenced by the high similarity between the single-ion and the masserstein images (Figure 7A,B). The segments obtained with intensity thresholding indicated that the lipid localized in the muscle tissue. However, the high-intensity segment was highly rugged (Figure 7C). Pixel-to-pixel variability impacted the spatial homogeneity of segments in the mouse cerebellum data set as well, although the effect was not as clearly visible as in the other two data sets, likely due to a smaller number of pixels (Supplementary Figure S14).

**Figure 7:**
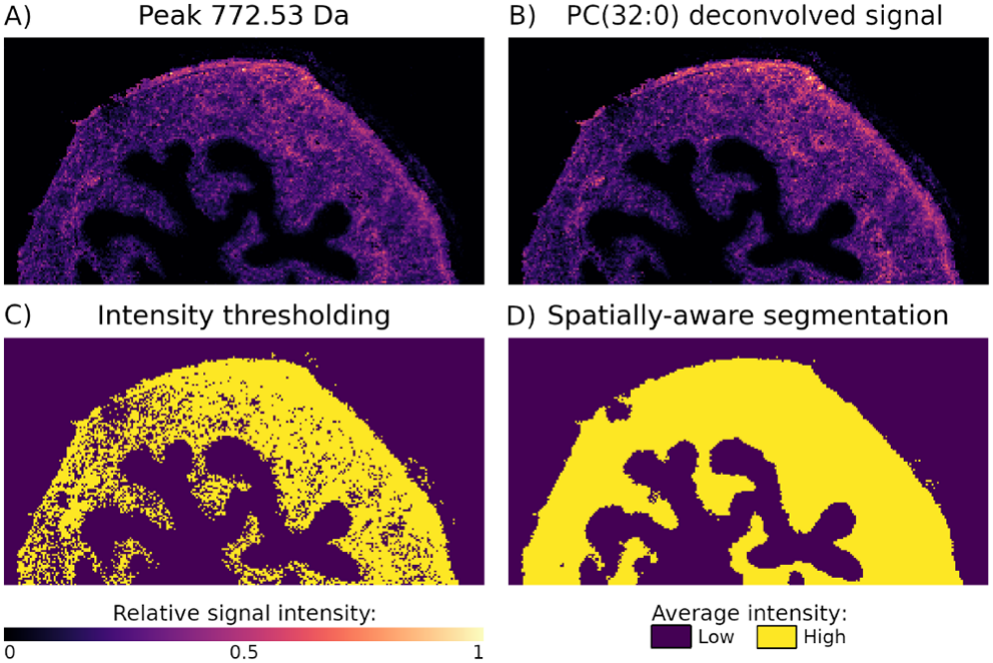
In the mouse bladder data set, pixel-to-pixel variability impacted the accuracy of segmentation independently from OIE. A) The single-ion image of the peak at 772.53 Da indicates that the analyte localizes in the muscle tissue. B) The spatialstein workflow annotated this peak with a lipid ion PC(32:0). The deconvolved ion image is identical to the single-ion image, indicating well-resolved isotopic envelopes and no interference due to OIE. C) Despite the lack of OIE interference, intensity thresholding failed to accurately identify the muscle tissue as the localization region. The high-intensity segment is rugged due to pixel-to-pixel variability of signal. D) A spatially aware segmentation with spatialDGMM mitigated the effect of pixel-to-pixel variability of the signal intensity and correctly identified the muscle tissue as the localization region.

These results demonstrate that pixel-to-pixel variability can highly impact the resulting segmentation even after OIE are resolved and the signals are accurately estimated. The random changes of signal between pixels are inherent to MSI data, rather than being a result of inaccurate estimation of the signal. These random changes cause pixels from different segments to have similar intensities, and pixels from a single region to have different intensities. This leads to spatially dispersed clusters which contain pixels from parts of different anatomical regions. Consequently, segmentation needs to be done with spatially aware methods regardless of how the signals of analytes are estimated.

### 3.5 Spatially Aware Segmentation of Deconvolved Ion Images Resulted in an Accurate Characterization of Tissues

In the simulated data set, spatially aware segmentation of deconvolved ion images resulted in spatially coherent clusters with a high degree of agreement with the true localization regions. For PC (38:1), PA(44:0) and PC(38:0) respectively, this approach correctly assigned 99%, 93% and 97% of pixels to low- and high-concentration regions (Figure 6, Supplementary Figure S15).

In the mouse bladder and cerebellum data sets, spatially aware segmentation improved the correspondence between segments and the underlying anatomical regions (Supplementary Figures S13, S14). Compared to a simple thresholding, spatialDGMM changed the pixel labels between high- and low-intensity segments for up to 20% of pixels in both data sets. On average, spatialDGMM changed the segment labels for 6% of pixels per lipid for the mouse cerebellum data set and 4% for the mouse bladder data set (Supplementary Figure S16).

For example, for the tentative PC(32:0) in the mouse bladder data set, the high-intensity segment obtained with spatialDGMM accurately highlighted the muscle tissue as the localization region (Figure 7D). In the mouse cerebellum data set, segmenting the deconvolved signals of the tentative PA (O-40:1) and SM(37:1) accurately matched their anatomical regions of concentration (the white matter for PA­(O-40:1) and the remaining tissue for SM(37:1); see Figure 5, cf. Figure 2, Supplementary Figure S14). This shows that a combination of OIE deconvolution and spatially aware segmentation can increase the amount of information that can be extracted from MSI data sets and can be used to identify regions of distinct chemical composition.

We note that, when applied to the nondeconvolved peak intensities of PA(44:0) in the simulated data set, the spatially aware approach performed worse than intensity thresholding (Supplementary Figure S15). In this case, the segmentation algorithm attempted to smooth out clusters that were highly misplaced due to OIE interference, which only decreased the agreement between clusters and the true localization regions. This result highlights the importance of a comprehensive segmentation workflow that correctly addresses all the inherent characteristics of the data, as a single incorrectly executed or omitted step of analysis can propagate throughout the workflow and result in incorrect segmentation.

### 3.6 Time- and Memory-Efficient Algorithms Allow for Processing Large Data Sets

As a final evaluation of spatialstein, we have used it to analyze a larger MSI data set with a higher mass resolution. The data set, referred to as *mouse brain*, generated as a part of the Lipid Brain Atlas project^45^ was downloaded from the METASPACE platform^44^ (ID 20220419_MouseBrain_female_217E_433x309_Att30_25μm). It has 133 808 pixels (pixel size 25*μm* x 25*μm*), obtained on an Orbitrap instrument with a resolving power R = 240 000 at *m*/*z* 200.

Since the data set was already centroided, we have skipped the preprocessing steps of spatialstein and proceeded directly to annotation in *thorough* mode on a sample of 40 000 randomly selected pixels. The annotation took approximately 1.5h on 16 CPUs of a personal laptop computer and detected 896 ions, 840 of which were above intensity threshold of 10^–9^. By comparison, METASPACE at FDR level 10% reported 43 unique molecular formulas matching our reference library of candidate ions, all of which were identified by spatiastein.

Deconvolution and estimation of proportions (*k*_*mixture*_ = 0.012, *k*_*components*_ = 0.016) took approximately 6h on 16 CPUs. The high resolving power of this data set was sufficient to resolve nearly all of the annotated ions. Nevertheless, we still have found three ions with indications of potential OIE interferences (Supplementary Figures S17, S18, S19).

For segmentation (*r* = 5, β = 8), we selected 274 ions which were detected in at least 10 000 pixels. The segmentation took approximately 20 min on 16 CPUs.

We note that since the annotation and deconvolution steps of spatialstein do not require loading the whole data set to the computer’s memory, the computational time is the main limiting factor for these steps. This can be easily mitigated by using more CPU cores for the analysis. Furthermore, annotation and deconvolution limit the memory requirements of downstream analyses by allowing the user to work with estimated proportion tables rather than imzML data files. For example, for the mouse brain data set, the original imzML file size was 2.4Gb, while the resulting proportion table was under 1Gb with no compression and 101Mb after zip compression.

## 4 Conclusions

Image segmentation methods help to discover distinct anatomical regions in MSI data sets without any prior knowledge of the data. However, in order to produce the correct regions of concentration of analytes, a segmentation workflow must correctly address the inherent structure of MSI data. In this work, we have developed a comprehensive workflow for annotation, linear deconvolution and segmentation of MSI data sets.

The spatialstein workflow overcomes two major challenges in MSI segmentation by leveraging recent developments in computational mass spectrometry: the masserstein package for linear deconvolution of spectra, which can be used for annotation of MSI data set and separation of overlapping isotopic envelopes, and the spatialDGMM algorithm, which combines pixels into spatially and chemically homogeneous segments. A combination of the two approaches produced a more biologically meaningful univariate segmentation of MSI data sets.

The implementation of the proposed workflow is available at https://github.com/mciach/spatialstein. The modular structure of the implementation, with each module addressing a specific part of the workflow, makes it applicable whole or in parts to other studies by users with intermediate knowledge of the programming languages Python (for annotation and deconvolution) and R (for segmentation). In particular, each module can be replaced with a solution preferred by the user (e.g., a different annotation method), or used on its own as a part of a pipeline (e.g., to add a deconvolution step to a multivariate segmentation pipeline). The workflow can be seamlessly integrated with additional preprocessing steps as needed. A further advantage of the modular structure is a greater degree of control over each step, which allows the users to check the intermediate results, adjust the parameters as necessary, and thus avoid errors propagating through the analysis.

We note that each step relies on algorithms that require the user to specify their parameters. In Supporting Information, we discuss the recommended practices in parameter estimation for selected steps. At a minimum, the users are advised to inspect the results of each step for a handful of different parameter values. The spatialstein workflow is specifically designed to give users control over intermediate results, allowing them to fine-tune parameter values during data analysis.

## Supplementary Material



## Notes

Detailed discussion of each step of the workflow and Supplementary Figures (PDF)

## References

[1] Buchberger A. R., DeLaney K., Johnson J., Li L. (2018). Mass spectrometry imaging:
a review of emerging advancements and future insights. Anal. Chem..

[2] Alexandrov T. (2012). MALDI imaging
mass spectrometry: statistical data analysis and current computational
challenges. BMC Bioinf..

[3] Verbeeck N., Caprioli R. M., Van de Plas R. (2020). Unsupervised
machine learning for
exploratory data analysis in imaging mass spectrometry. Mass Spectrom. Rev..

[4] Bemis K. D., Harry A., Eberlin L. S., Ferreira C. R., van de
Ven S. M., Mallick P., Stolowitz M., Vitek O. (2016). Probabilistic segmentation of mass spectrometry (MS) images helps
select important ions and characterize confidence in the resulting
segments. Mol. Cell. Proteomics.

[5] Hu H., Yin R., Brown H. M., Laskin J. (2021). Spatial segmentation of mass spectrometry
imaging data by combining multivariate clustering and univariate thresholding. Anal. Chem..

[6] Mas S., Torro A., Bec N., Fernández L., Erschov G., Gongora C., Larroque C., Martineau P., de Juan A., Marco S. (2019). Use of physiological information
based on grayscale images to improve mass spectrometry imaging data
analysis from biological tissues. Anal. Chim.
Acta.

[7] Mas S., Torro A., Fernández L., Bec N., Gongora C., Larroque C., Martineau P., De Juan A., Marco S. (2020). MALDI imaging
mass spectrometry and chemometric tools to discriminate highly similar
colorectal cancer tissues. Talanta.

[8] Jones E. A., Deininger S.-O., Hogendoorn P. C., Deelder A. M., McDonnell L. A. (2012). Imaging
mass spectrometry statistical analysis. J. Proteomics.

[9] Yang P., Zhang Z., Zhou B. B., Zomaya A. Y. (2010). A clustering based
hybrid system for biomarker selection and sample classification of
mass spectrometry data. Neurocomputing.

[10] Alexandrov T., Kobarg J. H. (2011). Efficient spatial
segmentation of large imaging mass
spectrometry datasets with spatially aware clustering. Bioinformatics.

[11] Alexandrov T., Becker M., Deininger S.-O., Ernst G., Wehder L., Grasmair M., Von Eggeling F., Thiele H., Maass P. (2010). Spatial segmentation
of imaging mass spectrometry data with edge-preserving image denoising
and clustering. J. Proteome Res..

[12] Watrous J. D., Alexandrov T., Dorrestein P. C. (2011). The evolving field of imaging mass
spectrometry and its impact on future biological research. J. Mass Spectrom..

[13] Guo D., Bemis K., Rawlins C., Agar J., Vitek O. (2019). Unsupervised
segmentation of mass spectrometric ion images characterizes morphology
of tissues. Bioinformatics.

[14] Peckner R., Myers S. A., Jacome A. S. V., Egertson J. D., Abelin J. G., MacCoss M. J., Carr S. A., Jaffe J. D. (2018). Specter: linear
deconvolution for targeted analysis of data-independent acquisition
mass spectrometry proteomics. Nat. Methods.

[15] De
Bruycker K., Krappitz T., Barner-Kowollik C. (2018). High performance
quantification of complex high resolution polymer mass spectra. ACS Macro Lett..

[16] Engler M. S., Crotty S., Barthel M. J., Pietsch C., Knop K., Schubert U. S., Bocker S. (2015). COCONUT—An Efficient
Tool
for Estimating Copolymer Compositions from Mass Spectra. Anal. Chem..

[17] Xiao K., Yu F., Fang H., Xue B., Liu Y., Tian Z. (2015). Accurate and
efficient resolution of overlapping isotopic envelopes in protein
tandem mass spectra. Sci. Rep..

[18] Höring M., Ejsing C. S., Krautbauer S., Ertl V. M., Burkhardt R., Liebisch G. (2021). Accurate quantification of lipid species affected by
isobaric overlap in Fourier-transform mass spectrometry. J. Lipid Res..

[19] Wang M., Huang Y., Han X. (2014). Accurate mass searching of individual
lipid species candidates from high-resolution mass spectra for shotgun
lipidomics. Rapid Commun. Mass Spectrom..

[20] Köfeler H. C., Ahrends R., Baker E. S., Ekroos K., Han X., Hoffmann N., Holčapek M., Wenk M. R., Liebisch G. (2021). Recommendations
for good practice in MS-based lipidomics. J.
Lipid Res..

[21] Cheng H., Miller D., Southwell N., Porcari P., Fischer J. L., Taylor I., Salbaum J. M., Kappen C., Hu F., Yang C. (2025). Untargeted
pixel-by-pixel metabolite ratio imaging
as a novel tool for biomedical discovery in mass spectrometry imaging. eLife.

[22] Hale O. J., Cooper H. J., Marty M. T. (2023). High-throughput
deconvolution of
native protein mass spectrometry imaging data sets for mass domain
analysis. Anal. Chem..

[23] Glodek, A. Fuzzy-Inference System for Isotopic Envelope Identification in Mass Spectrometry Imaging Data. In Bioinformatics and Biomedical Engineering: 9th International Work-Conference; Springer-Verlag: Berlin, Heidelberg, 2022, Vol: 13347, pp. 119–132. DOI: 10.1007/978-3-031-07802-6_10.

[24] Majewski, S. ; Ciach, M. A. ; Startek, M. ; Niemyska, W. ; Miasojedow, B. ; Gambin, A. The wasserstein distance as a dissimilarity measure for mass spectra with application to spectral deconvolution. In. 18th International Workshop On Algorithms In Bioinformatics; GmbH, 2018, Vol: 113, pp. 1-25. DOI: 10.4230/LIPIcs.WABI.2018.25.

[25] Ciach M. A., Miasojedow B., Skoraczyński G., Majewski S., Startek M., Valkenborg D., Gambin A. (2025). Masserstein: Linear regression of
mass spectra by optimal transport. Rapid Commun.
Mass Spectrom..

[26] Domżał B., Nawrocka E. K., Gołowicz D., Ciach M. A., Miasojedow B., Kazimierczuk K., Gambin A. (2024). Magnetstein: An Open-Source Tool
for Quantitative NMR Mixture Analysis Robust to Low Resolution, Distorted
Lineshapes, and Peak Shifts. Anal. Chem..

[27] Seifert N. A., Prozument K., Davis M. J. (2021). Computational optimal transport for
molecular spectra: The fully discrete case. J. Chem. Phys..

[28] Seifert N. A., Prozument K., Davis M. J. (2022). Computational optimal transport for
molecular spectra: The semi-discrete case. J.
Chem. Phys..

[29] Seifert N. A., Prozument K., Davis M. J. (2023). Computational optimal transport for
molecular spectra: The fully continuous case. J. Chem. Phys..

[30] Bochenek M., Ciach M. A., Smeets S., Beckers O., Vanderspikken J., Miasojedow B., Domżał B., Valkenborg D., Maes W., Gambin A. (2024). An Automated
Analysis of Homocoupling
Defects Using MALDI-MS and Open-Source Computer Software. J. Am. Soc. Mass Spectrom..

[31] Łącki M.
K., Startek M., Valkenborg D., Gambin A. (2017). IsoSpec: Hyperfast
Fine Structure Calculator. Anal. Chem..

[32] Guo A., Chen Z., Li F., Luo Q. (2023). Delineating regions
of interest for mass spectrometry imaging by multimodally corroborated
spatial segmentation. GigaScience.

[33] Bemis K. A., Föll M. C., Guo D., Lakkimsetty S. S., Vitek O. (2023). Cardinal v.3: a versatile open-source software for mass spectrometry
imaging analysis. Nat. Methods.

[34] Eberlin L. S., Tibshirani R. J., Zhang J., Longacre T. A., Berry G. J., Bingham D. B., Norton J. A., Zare R. N., Poultsides G. A. (2014). Molecular
assessment of surgical-resection margins of gastric cancer by mass-spectrometric
imaging. Proc. Nal. Acad. Sci..

[35] Dexter A., Race A. M., Steven R. T., Barnes J. R., Hulme H., Goodwin R. J., Styles I. B., Bunch J. (2017). Two-phase and graph-based
clustering methods for accurate and efficient segmentation of large
mass spectrometry images. Anal. Chem..

[36] Zhang W., Claesen M., Moerman T., Groseclose M. R., Waelkens E., De Moor B., Verbeeck N. (2021). Spatially
aware clustering
of ion images in mass spectrometry imaging data using deep learning. Anal. Bioanal. Chem..

[37] Haug K., Cochrane K., Nainala V. C., Williams M., Chang J., Jayaseelan K. V., O’Donovan C. (2020). MetaboLights:
a resource evolving
in response to the needs of its scientific community. Nucleic Acids Res..

[38] Bond N. J., Koulman A., Griffin J. L., Hall Z. (2017). massPix: an R package
for annotation and interpretation of mass spectrometry imaging data
for lipidomics. Metabolomics.

[39] Perez-Riverol Y., Bai J., Bandla C., García-Seisdedos D., Hewapathirana S., Kamatchinathan S., Kundu D. J., Prakash A., Frericks-Zipper A., Eisenacher M., Walzer M., Wang S., Brazma A., Vizcaíno J. A. (2022). The PRIDE database resources in 2022:
a hub for mass
spectrometry-based proteomics evidences. Nucleic
Acids Res..

[40] Römpp A., Guenther S., Schober Y., Schulz O., Takats Z., Kummer W., Spengler B. (2010). Histology by mass spectrometry: label-free
tissue characterization obtained from high-accuracy bioanalytical
imaging. Angew. Chem. Int., Ed..

[41] Clinical metabolomics: methods and protocols, Giera, M. ; Sánchez-López, E. ; Springer, 2025. 10.1007/978-1-0716-4116-3_1 39354298

[42] Sud M., Fahy E., Cotter D., Brown A., Dennis E. A., Glass C. K., Merrill A. H., Murphy R. C., Raetz C. R., Russell D. W. (2007). Lmsd: Lipid maps structure
database. Nucleic Acids Res..

[43] Kibbe R. R., Muddiman D. C. (2024). Quantitative mass
spectrometry imaging (qMSI): a tutorial. J.
Mass Spectrom..

[44] Palmer A., Phapale P., Chernyavsky I., Lavigne R., Fay D., Tarasov A., Kovalev V., Fuchser J., Nikolenko S., Pineau C. (2017). FDR-controlled
metabolite annotation for high-resolution
imaging mass spectrometry. Nat. Methods.

[45] Bassini, L. F. ; Schede, H. H. ; Capolupo, L. ; Alieh, L. H. A. ; Venturi, F. ; Valente, A. ; Droin, C. ; Banos, D. T. ; Khven, I. ; Asirim, E. Z. , The lipidomic architecture of the mouse brain. bioRxiv. 2025. 10.1101/2025.10.13.682018

