# Supplementary material for: spatialstein: An Open-Source Workflow for Annotation, Deconvolution, and Spatially Aware Segmentation of Mass Spectrometry Imaging Data
Source: Anal Chem. 2026 Jan 2;98(1):364–75. doi: 10.1021/acs.analchem.5c04737 (PMC12809658; doi:10.1021/acs.analchem.5c04737)
Supplement: Supplementary file 1 [file ac5c04737_si_001.pdf]

Supporting Information

spatialstein: an open-source workflow for annotation, deconvolution and spatially-aware segmentation of mass spectrometry imaging data

Michał Aleksander Ciach,[*,†,‡,¶] Dan Guo,[§] Kylie Ariel Bemis,[§] Dirk Valkenborg,[¶] Olga Vitek,[§] and Anna Gambin[‡]

†*Department of Applied Biomedical Science, Faculty of Health Sciences, University of Malta, Msida, MSD 2080, Malta*

‡*Faculty of Mathematics, Informatics and Mechanics, University of Warsaw, Warsaw, 02-097, Poland*

¶*Data Science Institute, Hasselt University, Diepenbeek, 3590, Belgium*

§*Khoury College of Computer Sciences and Barnett Institute, Northeastern University, Boston, MA 02115, USA*

E-mail: michal.ciach@um.edu.mt

Contents

Designing an MSI data segmentation workflow **S3**

Pre-processing S3

Peak assignment S4

Deconvolution and quantification S5

Segmentation S7

General remarks S8

Supplementary Figures **S10**

Supplementary Information

Designing an MSI data segmentation workflow

A segmentation workflow of MSI datasets needs to be composed of several steps, and a correct implementation of each step is necessary to obtain correct results. Errors at any step can propagate throughout the workflow and distort the end results. In this section, we discuss good practices and caveats in designing and using a segmentation workflow such as `spatialstein`. The purpose of this section is to help new users obtain accurate results and avoid potential sources of errors. We also discuss advantages and disadvantages of alternative approaches to selected steps of the workflow.

Pre-processing

The first necessary step of any workflow is an adequate pre-processing of the dataset, which typically includes centroiding and normalization. Picking a centroiding procedure needs to address a trade-off between computational complexity and accuracy. For MSI datasets, highly accurate procedures such as wavelet transforms[1] may be prohibitively computationally expensive. Consequently, simplified procedures may need to be used, such as detecting local maxima and integrating the peaks within full width at half maximum. However, when OIEs are present, this approach may result in significant displacement of peaks and in merging multiple peaks into one centroid (see examples in Suppl. Figs. S2, S10). In this case, subsequent steps need to use software which is robust against peak displacements, such as `masserstein`.

Normalizing spectra is used to make the signals more comparable between spectra by addressing issues such as systematic bias and variance in the ionization efficiency between pixels.[2] The most accurate normalization method uses a standard analyte which is present in the same concentration throughout the sample. In the absence of a standard, a common

normalization method is TIC (total ion current), which equalizes the total signal in all spectra.[3] A downside of TIC normalization is that it removes the information about the overall concentration of lipids in different tissues. Because of this, choosing if and how to normalize spectra needs to address a trade-off between correcting for biases and information loss, which may be different for different datasets. In our examples, TIC normalization improved the segmentation of the mouse and cerebellum datasets, but decreased its accuracy for the simulated dataset which did not contain extensive variances or biases in ionization intensities. Therefore, it is a good practice to inspect the results of several normalization procedures to identify the optimal approach for the data at hand, or to add a standard when preparing the dataset.[2]

After normalizing the pixel spectra we restricted their m/z range to 700 Da to 900 Da in order to improve the computational efficiency of our workflow and focus on the signals of the analytes of interest. We remark that the order of these two operations is important for accurate signal quantification, as normalizing the spectra after restricting them to the mass range of interest, rather than before, may lose information about the total ion current and artificially inflate the trace lipid signals in the background. This may in turn highly influence the apparent spatial distribution of analytes and result in incorrect results in downstream analysis.

Peak assignment

The second step of the workflow is annotation (a.k.a. peak assignment), which assigns molecular formulas to peaks. In the absence of tandem MS data, one of the possible approaches to this step is to annotate the average spectrum of the dataset (obtained by summing all pixel spectra). Average spectra are commonly used to select peaks of interest in MSI data analysis.[4] However, our results indicate that aggregating thousands of individual pixel spectra to create an average one can hide important signals and lead to false negative results, especially in the presence of OIEs (see Supplementary Fig. S11 for examples of "hidden lipids" in the

average spectra of the mouse cerebellum dataset). To overcome the problems caused by annotating aggregated spectra, we adopted a reverse strategy, in which we first annotate all pixel spectra and then aggregate the annotations. This strategy allowed us to obtain 180 and 209 tentative annotations in the mouse bladder and mouse cerebellum datasets, respectively. Using the same software with the same parameters to annotate the average spectrum resulted in 31 and 128 tentative annotations, confirming that many ions are lost when annotating aggregated spectrum. The *annotate, then average* strategy has an additional advantage in that it allows to refine the annotation by using the spatial information, e.g. by selecting only the analytes which are detected in a sufficient number of pixels.

While the *annotate, then average* strategy has considerable advantages over annotating an aggregated spectrum, choosing an appropriate annotation approach needs to address a trade-off between accuracy and computational complexity, similar to selecting a centroiding procedure. We also remark that while tentative annotations obtained by matching isotopic envelopes or accurate masses offer a valuable starting point for statistical analyses and exploratory studies, they have a relatively high risk of mistaking similar ions due to the limitations of mass accuracy and resolving power, and therefore need to be verified with other methods if an accurate assignment of lipid ions is important for the research question at hand.

Deconvolution and quantification

The third step of our workflow is the quantification of the signals corresponding to the annotated analytes in each pixel. This step provides the spatial distributions of the analytes. While in this work we have focused mostly on the need to use a method capable of resolving overlapping envelopes, several other considerations need to be addressed when choosing the appropriate method of computational quantification of signals. When signals are estimated by a linear deconvolution, one of the most important considerations is checking the validity of the model assumptions, i.e. whether theoretically predicted spectra properly model the

experimental ones. Common model violations include the aforementioned peak shifting due to centroiding of overlapping signals, incorrect calibration of the spectra causing a systematic shift of m/z values, missing signals due to insufficient ion statistics (in the context of lipidomics, this is especially common for the heavier isotopic peaks - while a typical lipid has up to 20 theoretical peaks, only the first three or four are typically visible in the spectrum), and signals not accounted for by the model (e.g. signals of contaminants or other ions overlapping with the ions of interest).

A less intuitive model violation that we encountered is the apparent lack of the ^{41}K isotope in the mouse bladder dataset, in which lipids are ionized with a potassium adduct.[5] While we do not have an explanation as to why lipid ions would be ionized only with a ^{40}K adduct and not a ^{41}K one, the theoretically predicted ^{41}K peak is missing in the spectra (Fig. S20). All these model violations need to be accounted for either by an appropriate preprocessing or the use of robust software in order to avoid biasing the results. Furthermore, we stress that in order to detect model violations, it is crucial to visually inspect the fitted models and the predicted isotopic distributions of the annotated ions.

We note that, even though our workflow uses the same software both for the annotation and estimation steps, separating those steps is necessary for an accurate segmentation. Using `masserstein` to directly quantify the 3523 lipid ions retrieved from the LIPID MAPS data base produced unreliable results due to two reasons. First, ions with nearly identical isotopic envelopes compete for the same signals, and the resulting estimation is highly influenced by random factors such as ion statistics or mass inaccuracies. Second, the annotation and the quantification steps have different requirements: the annotation step needs to prioritize accurate mass matching, while the quantification step needs to prioritize using all of the the signal of an ion. For this reason, in the annotation step we use more stringent κ parameters than in the quantification step. The parameters for the annotation step require that the expected distance between predicted and observed peaks is less than 0.005 Da, while the parameters for the quantification step allow deviations up to 0.01 Da. Excessive values of

the κ parameter in the annotation stage result in multiple false positive annotations, while insufficient values in the quantification step leads to an underestimation of the lipid signal.

Segmentation

The `spatialDGMM` algorithm, used in this work as a spatially-aware segmentation method, has two parameters which control the strength of the spatial smoothing. The radius parameter, r, controls which pixels are considered "neighbors". For each pixel, `spatialDGMM` calculates a weighted average of the posterior probability of segment assignment with the probabilities of pixels within the radius r. The β parameter controls the relative strength of smoothing. The larger the β, the more influence of the neighboring pixels on the assignment of a given pixel.

These two parameters have different impacts on the resulting segmentation. Large values of the radius r are suitable for discovering larger anatomical regions. This can lead to a loss of smaller details in the segmentation. In turn, lower values of the radius allow for less smoothing, but can discover more detailed anatomical regions. In contrast, the β parameter controls the amount of spatial smoothing within the fixed radius. While small values of β effectively disable spatial smoothing, large values of β cause the algorithm to prioritize spatial relations of pixels over the chemical information contained in their spectra, potentially leading to a loss (or merging) of distinct anatomical regions and/or numerically unstable results.

Both parameters need to be tuned in order to strike a balance between spatial smoothing and the loss of information. Accordingly, we recommend using the lowest values of parameters which result in sufficiently spatially coherent clusters. In this work, we first tuned the radius parameter to avoid losing small anatomical regions, followed by tuning the β parameter to obtain smooth clusters.

General remarks

All of the aforementioned steps rely on the correct settings of algorithm parameters. It is highly advised not to rely on the default parameter values of software, as the correct values might vary between instruments and tissues. Unfortunately, in most cases there are no methods to calculate the ”true” parameter values a priori. Thereore, users should always inspect and compare the results of each step for different parameter values and use measurable benchmarks whenever possible (e.g. testing whether known lipid ions are correctly detected, whether different quantification methods agree on simple cases, whether known tissues are correctly delineated etc). While most of the software in our workflow is stable for a relatively broad range of parameters (meaning that highly precise values are not necessary), a gross misspecification of parameters results in incorrect results.[6]

References

(1) Du, P.; Kibbe, W. A.; Lin, S. M. Improved peak detection in mass spectrum by incorporating continuous wavelet transform-based pattern matching. *bioinformatics* **2006**, *22*, 2059–2065.

(2) Buchberger, A. R.; DeLaney, K.; Johnson, J.; Li, L. Mass spectrometry imaging: a review of emerging advancements and future insights. *Analytical Chemistry* **2018**, *90*, 240.

(3) Kibbe, R. R.; Muddiman, D. C. Quantitative mass spectrometry imaging (qMSI): a tutorial. *Journal of Mass Spectrometry* **2024**, *59*, e5009.

(4) Murta, T.; Steven, R. T.; Nikula, C. J.; Thomas, S. A.; Zeiger, L. B.; Dexter, A.; Elia, E. A.; Yan, B.; Campbell, A. D.; Goodwin, R. J.; others Implications of peak selection in the interpretation of unsupervised mass spectrometry imaging data analyses. *Analytical Chemistry* **2021**, *93*, 2309–2316.

(5) Römpp, A.; Guenther, S.; Schober, Y.; Schulz, O.; Takats, Z.; Kummer, W.; Spengler, B. Histology by mass spectrometry: label-free tissue characterization obtained from high-accuracy bioanalytical imaging. *Angewandte Chemie International Edition* **2010**, *49*, 3834–3838.

(6) Bochenek, M.; Ciach, M. A.; Smeets, S.; Beckers, O.; Vanderspikken, J.; Miasojedow, B.; Domżał, B.; Valkenborg, D.; Maes, W.; Gambin, A. An Automated Analysis of Homocoupling Defects Using MALDI-MS and Open-Source Computer Software. *Journal of the American Society for Mass Spectrometry* **2024**, *35*, 2366–2375.

Supplementary Figures

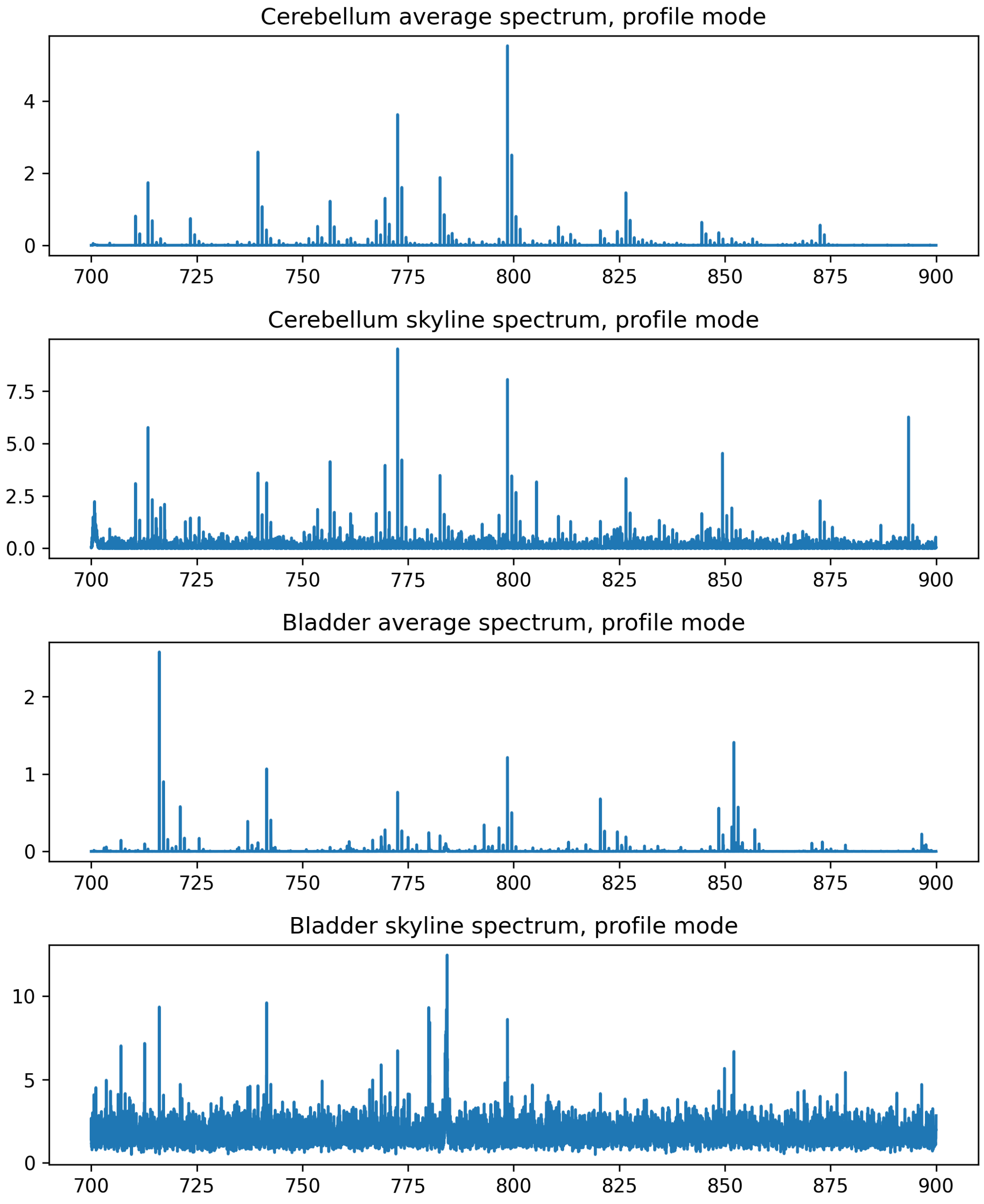

Supplementary Figure S1: Average and skyline spectra of the mouse cerebellum and mouse bladder mass spectrometry datasets. Average spectra show the signal averaged over all pixels and reflect the overall composition of the dataset. Skyline spectra show the maximum signal over all pixels, and reflect compounds concentrated in small regions of the datasets, as well as contaminants, outliers and background noise. Comparing the two types of spectra per dataset also reflects the degree of pixel-to-pixel variability.

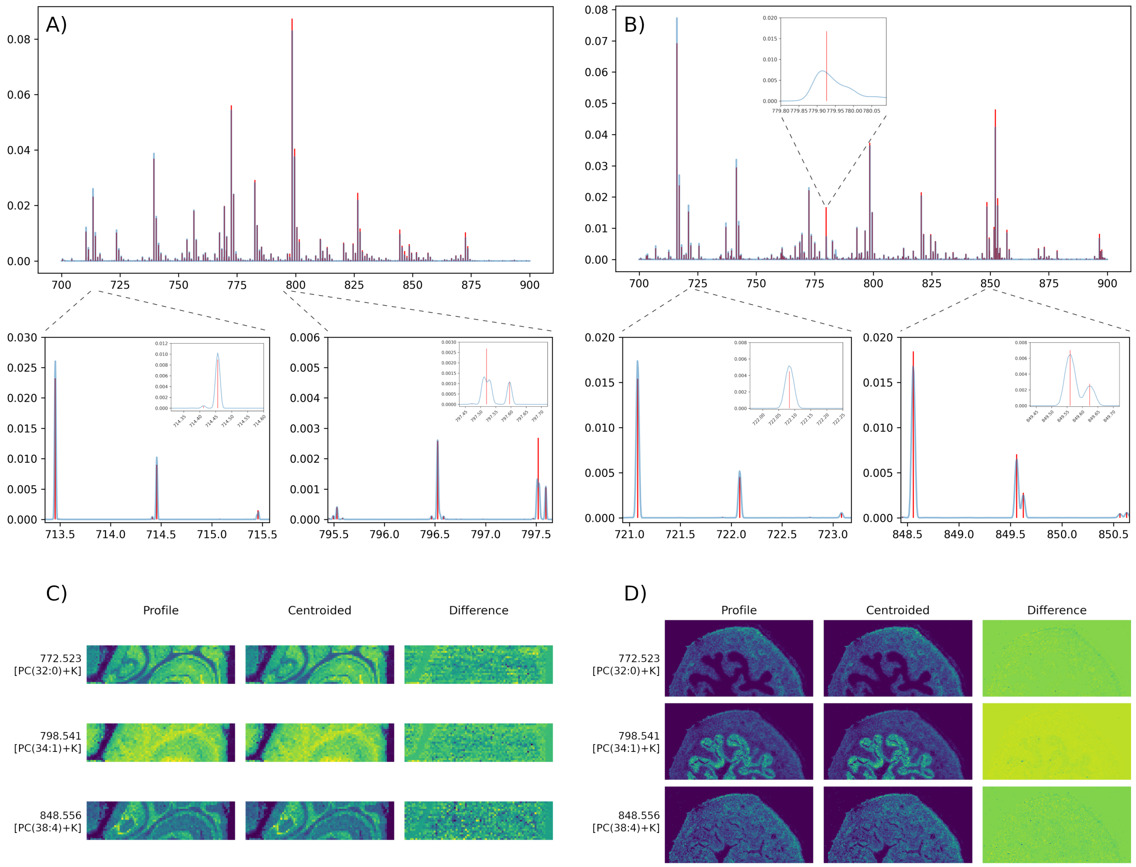

Supplementary Figure S2: Verification of the correctness of centroiding procedure. A) The average spectrum of the mouse cerebellum dataset in profile and centroided mode, and two zoomed in fragments. An extensive overlap of two peaks at 797.53 Da (zoomed in) caused them to be detected as a single centroid located between them. B) The average spectrum of the mouse bladder dataset in profile and centroided mode, and two zoomed in fragments. Overlapping peaks at 849.55 Da and 849.63 Da were correctly identified as two centroids thanks to a sufficient separation of the signals. In A), B) the profile spectra were rescaled so that the intensities are comparable between the profile and centroided modes. Note that, because the width of the peaks in profile mode increases with the m/z value, the height of centroided peaks is below the profile spectrum for low m/z values and above it for high m/z values; as a consequence, peak heights in profile mode are not correct estimates of ion abundances, necessitating integration of their areas. C) Single peak images of selected lipid ions obtained from spectra in centroided and profile modes in the mouse cerebellum dataset. D) Single peak images of selected lipid ions obtained from spectra in centroided and profile modes in the mouse bladder dataset. Similar distributions of lipids between datasets obtained from the two modes in C), D) confirm the correctness of centroiding.

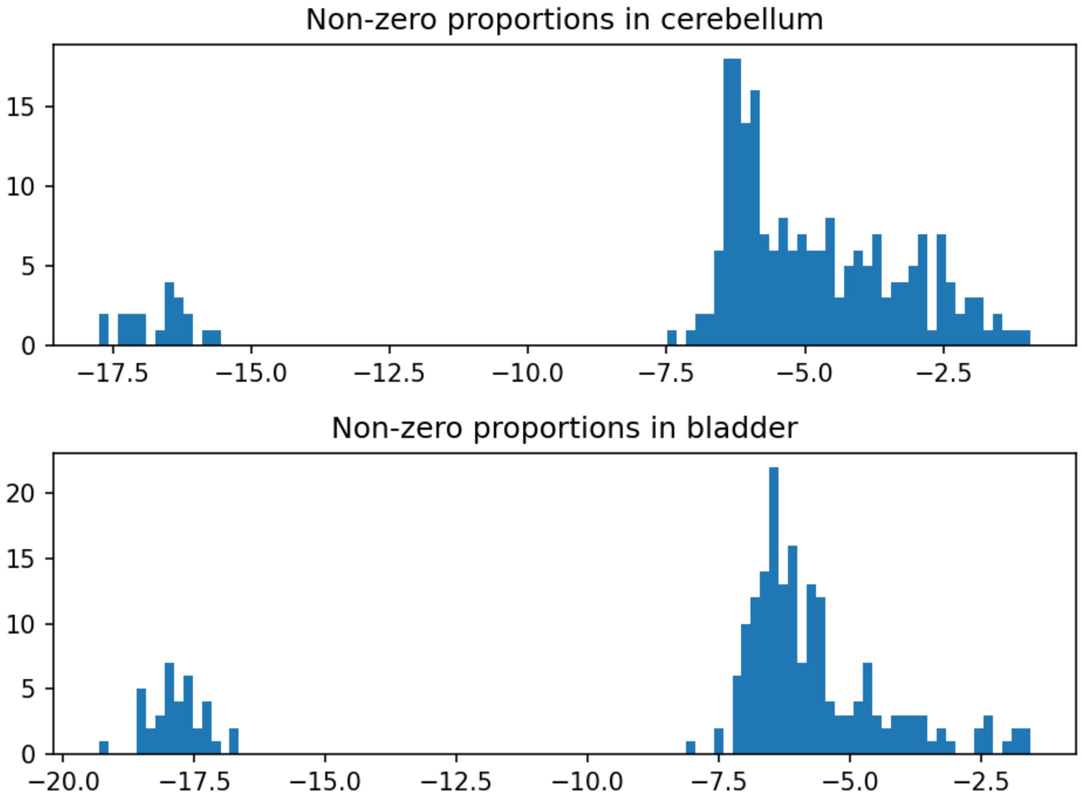

Supplementary Figure S3: Histograms of total lipid proportions estimated in the annotation step. For each annotated molecular formula, the proportions estimated with `masserstein` in the annotation step were summed over all pixels. The proportions estimated in the annotation step are a rough indication of the presence or absence of an ion, because the software parameters in this step are tuned to maximize the mass accuracy of peaks rather than the accuracy of quantification. The separation between the two distribution suggests that low-intensity signals correspond to lipid isotopic envelopes fitted to background noise, while high-intensity signals correspond to isotopic envelopes fitted to genuine signals of the same or similar ion. We refer to our annotations as tentative due to the possibility of envelopes being fitted to signals of ions with a very similar chemical composition.

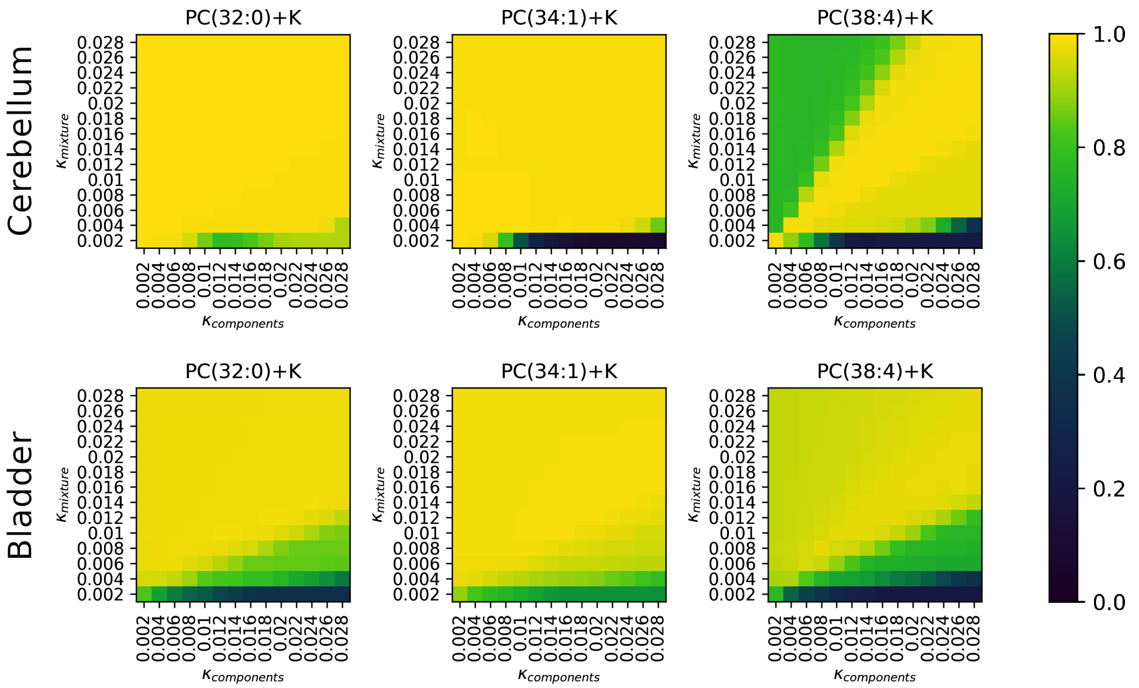

Supplementary Figure S4: Heatmaps of correlation between signals estimated with `masserstein` and monoisotopic peak intensities for selected three lipids without OIE interference. The correlation of signals was estimated from a random sample of 1000 pixels in each dataset.

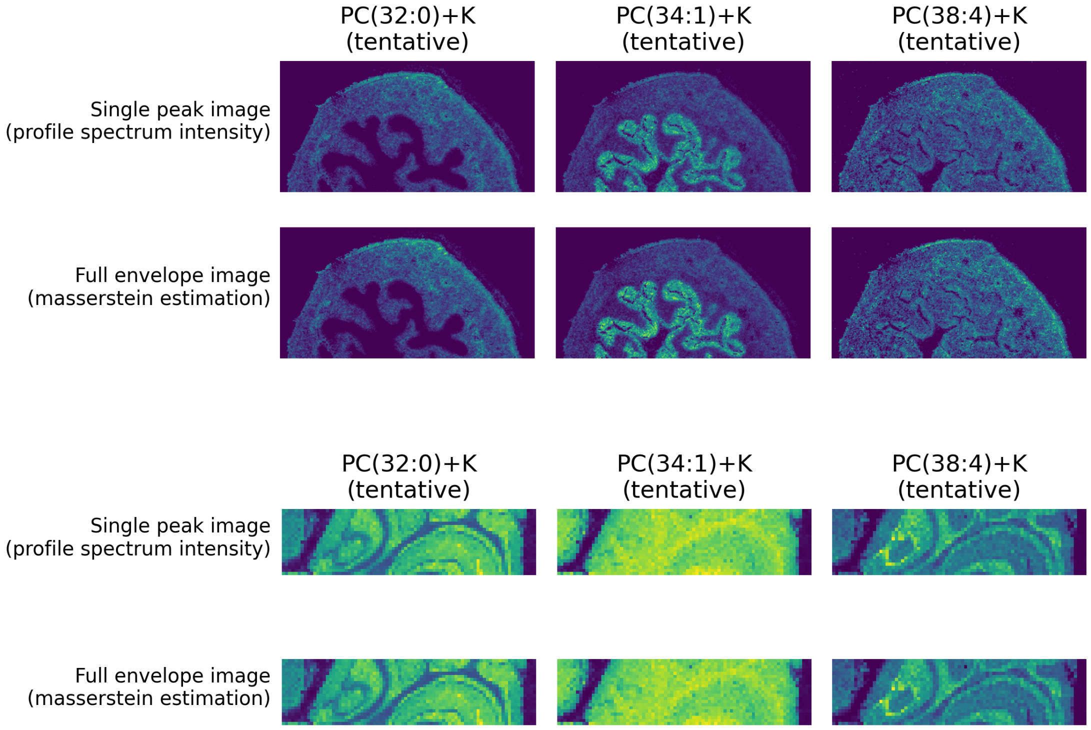

Supplementary Figure S5: Verification of the correctness of `masserstein` estimation on lipids for which a manual inspection of spectra indicated no interference due to overlapping isotopic envelopes. For such lipids, `masserstein` is expected to return results similar to the monoisotopic peak intensity.

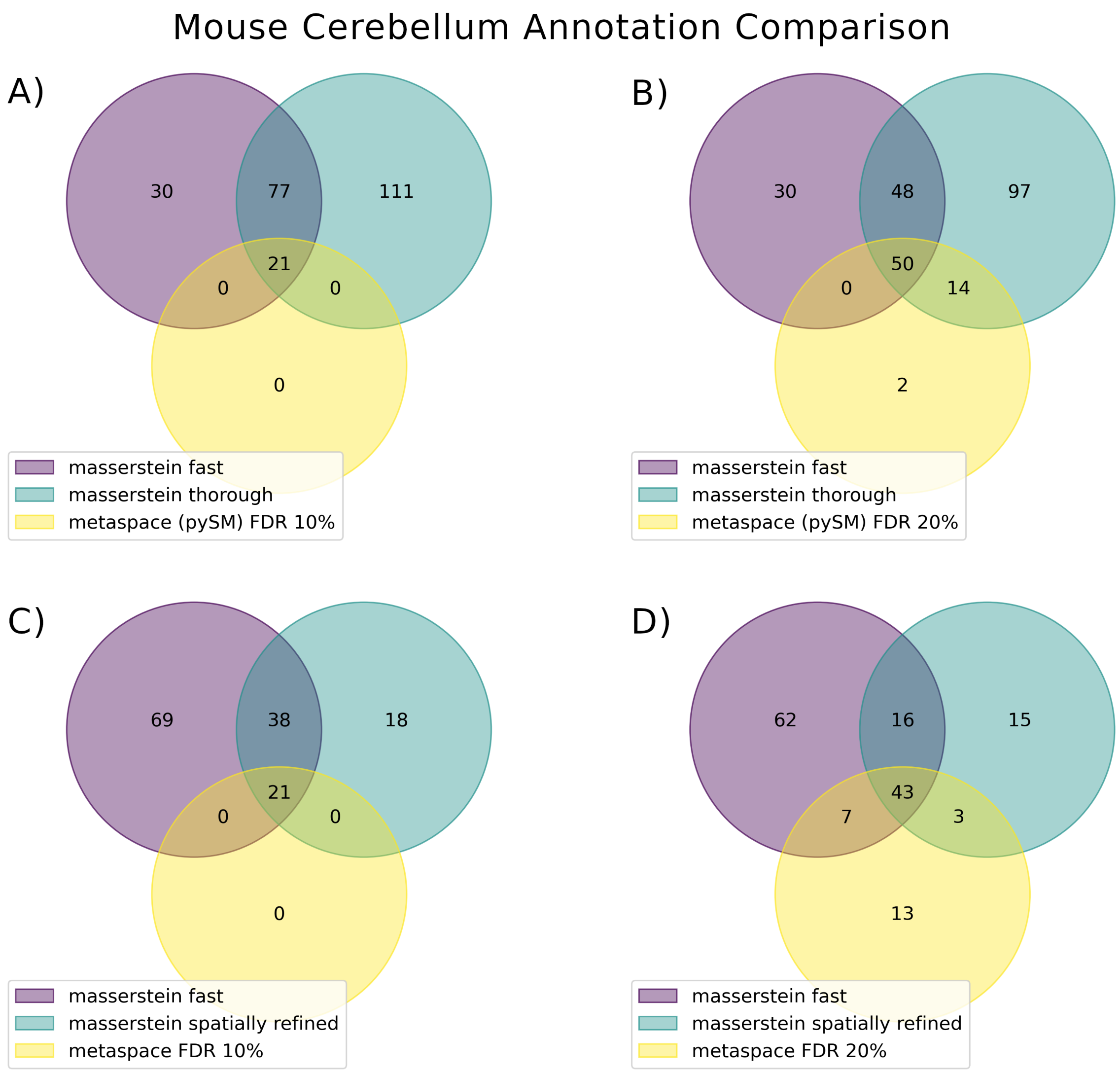

Supplementary Figure S6: A comparison of annotation results of the Mouse Cerebellum dataset for five annotation strategies: `spatialstein` based on the `masserstein` algorithm in "fast" mode (A, B, C, D), "thorough" mode (A, B), "thorough" mode with spatial filter (ion present in at least 400 pixels; C, D), and pySM results downloaded from METASPACE for FDR level 10% (A, B) and 20% (C, D).

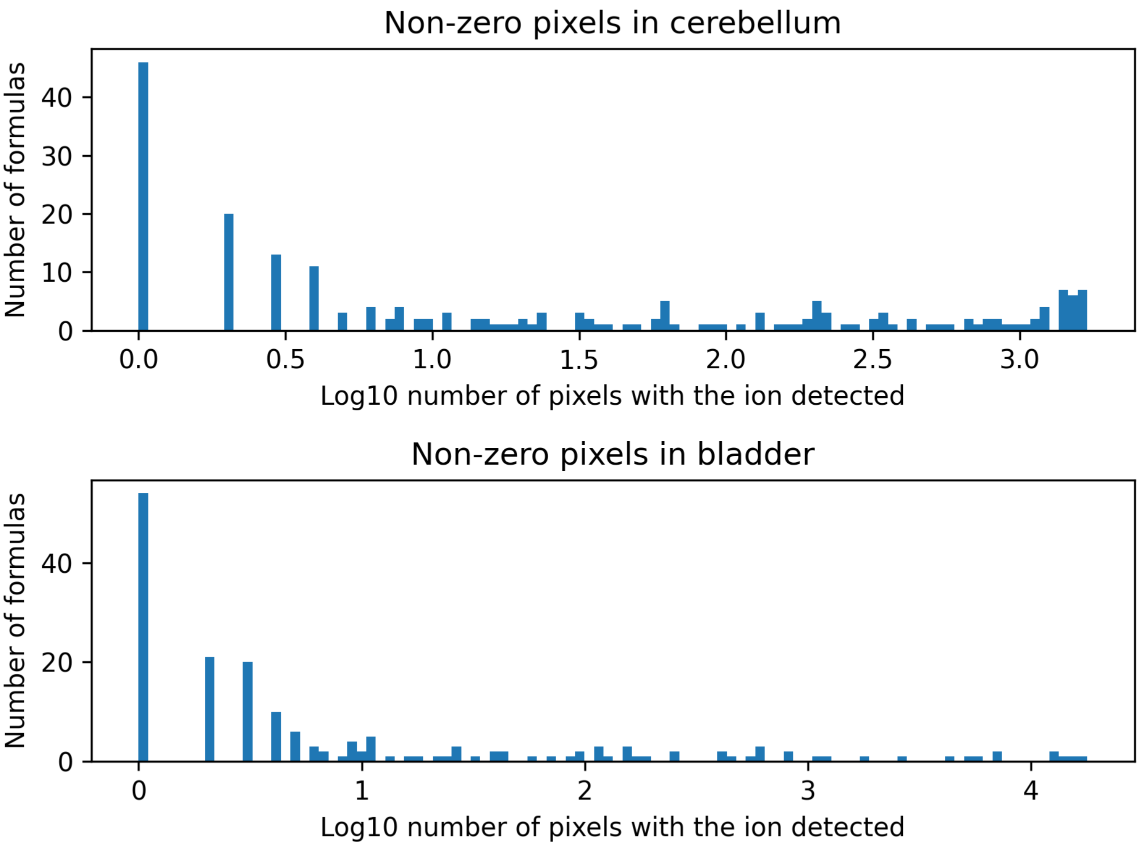

Supplementary Figure S7: Histograms of the log10 number of pixels in which lipid ions were assigned a non-zero signal in the linear deconvolution phase of the workflow.

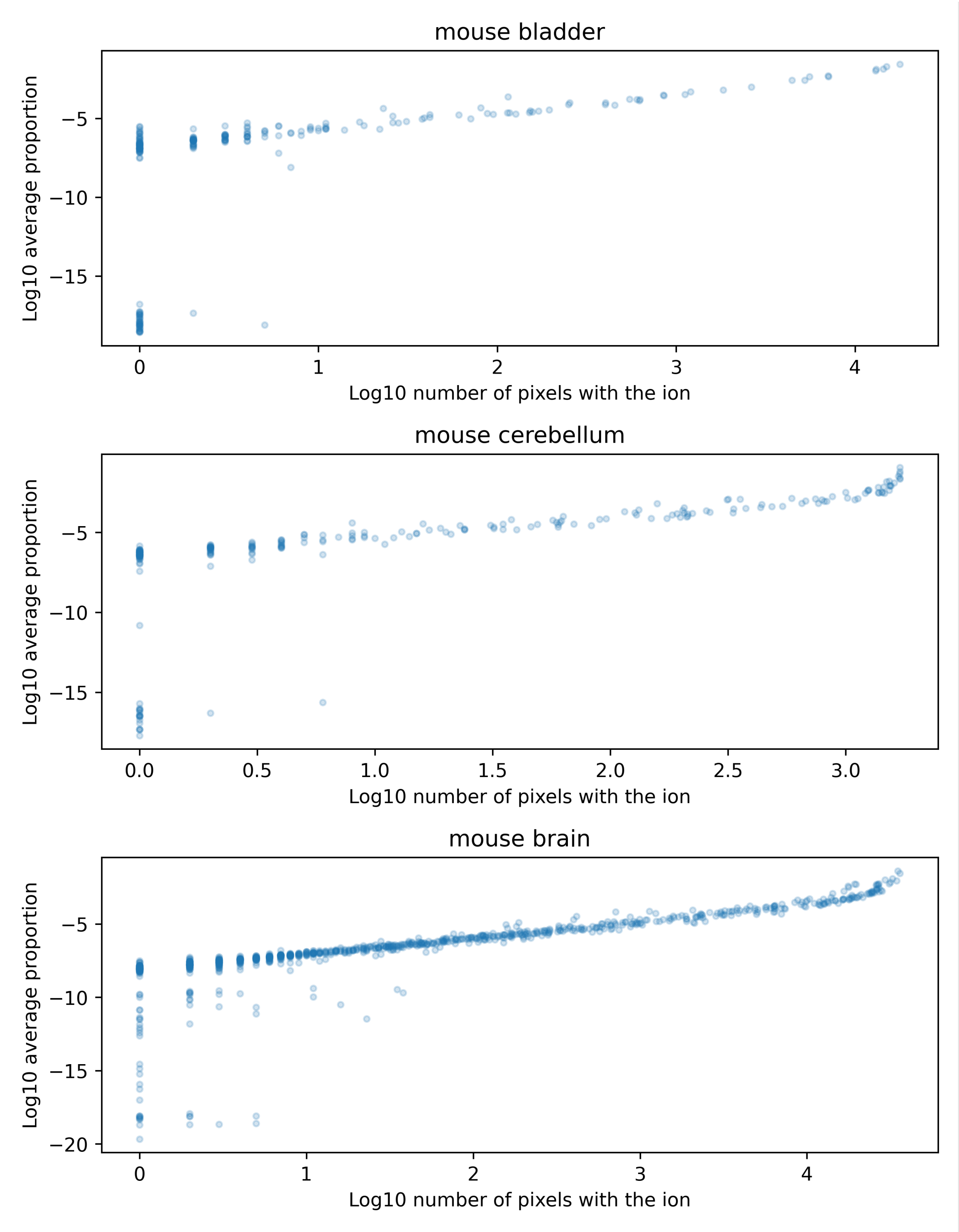

Supplementary Figure S8: The relation between the number of pixels in which a lipid ion was detected and the overall average estimated proportion of the ion in the MSI dataset.

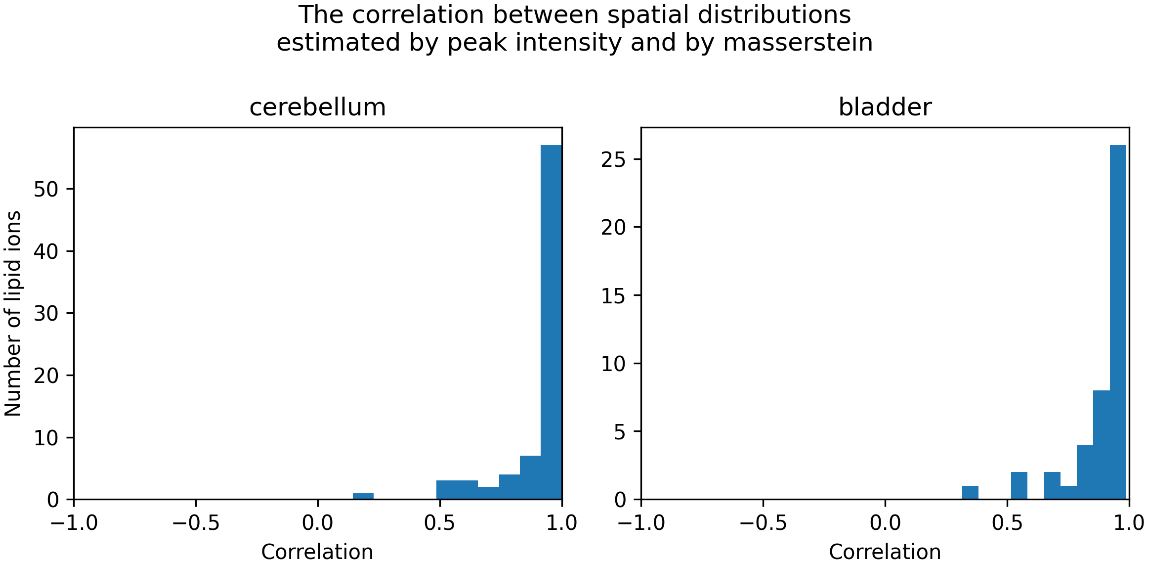

Supplementary Figure S9: The correlation of lipid proportions estimated by the monoisotopic peak intensity and by `masserstein`. In the cerebellum dataset, the correlation was calculated for lipids present in at least 400 pixels. In the bladder dataset, the correlation was calculated for lipids present in at least 1000 pixels.

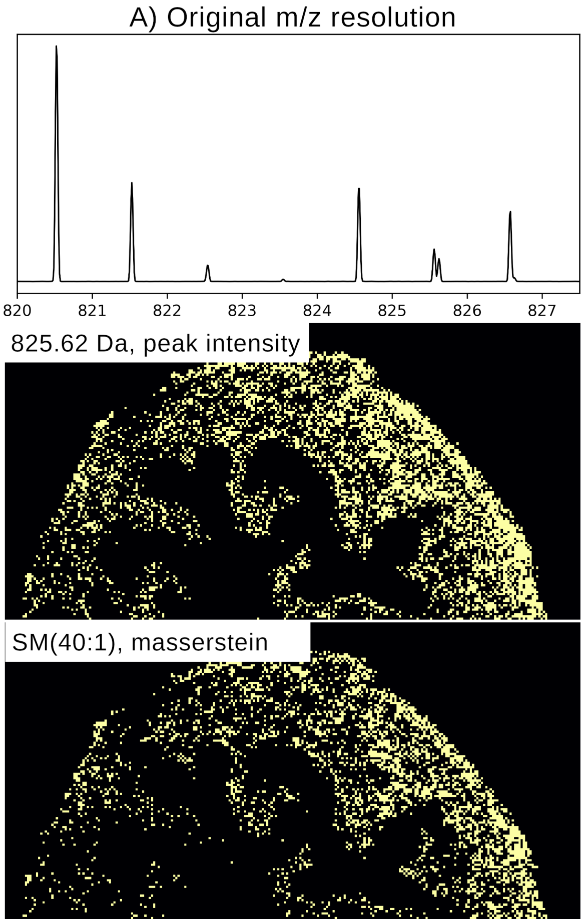

Supplementary Figure S10: The result of a computational experiment to verify that `masserstein` correctly estimates the signals of analytes with overlapping isotopic envelopes in the mouse bladder MSI dataset. A) A fragment of the average spectrum of the mouse bladder MSI dataset, and the segmentation of the peak intensity at 852.62 Da and of the signal of the tentative SM(40:1) estimated with `masserstein`. The segmentations are nearly identical due to a sufficient separation of the signals in the pixel spectra. B) The low m/z resolution average spectrum shows the result of applying a Gaussian filter to the pixel spectra of the dataset to broaden their signals and lower their resolution. The broadening of the signals results in merging two peaks at 825.62 Da. As a result, the peak intensity corresponds to a mixture of the spatial distributions of two analytes. However, estimating the signal with `masserstein` returns almost identical result for both the high-resolution and low-resolution datasets, confirming a succesful separation of overlapping signals.

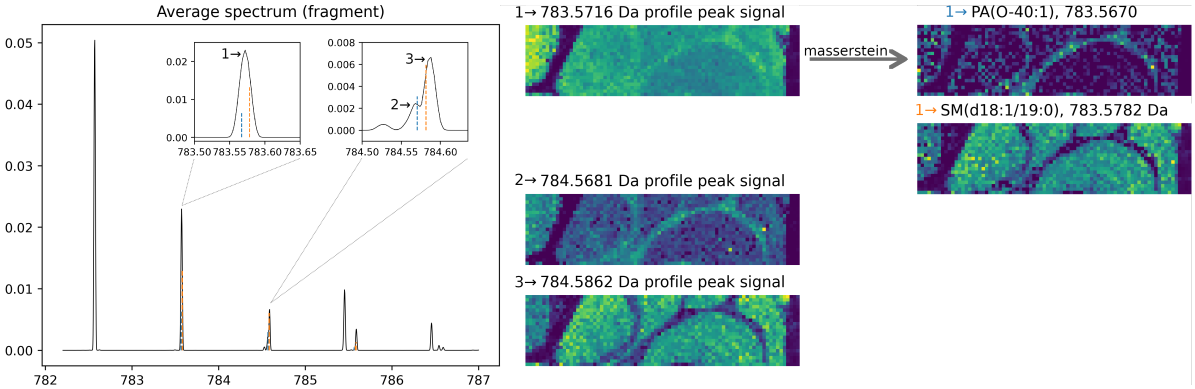

Supplementary Figure S11: An example of an ion image distorted due to overlapping isotopic envelope interference in the mouse cerebellum dataset. The ion image of the peak [1→783.5716 Da] suggests that the ion is distributed throughout the tissue. However, fitting theoretical isotopic envelopes with `masserstein` suggests that this peak is composed of at least two lipid ions: A phosphatidic acid PA(O-40:1), 783.5670 Da, and a sphingomyelin SM(19:0), 783.5782 Da. The two lipid ions have complementary spatial distributions, with PA(O-40:1) located specifically in the white matter and SM(19:0) specifically outside of it. The ion image of the peak [1→783.5716 Da] is therefore a combination of two complementary ion images. Inspecting the ion images of the isotopic peaks [2→784.5681 Da] and [3→784.5862] confirms the presence of two ions with complementary spatial distribution, corroborating the results of `masserstein`-based annotation.

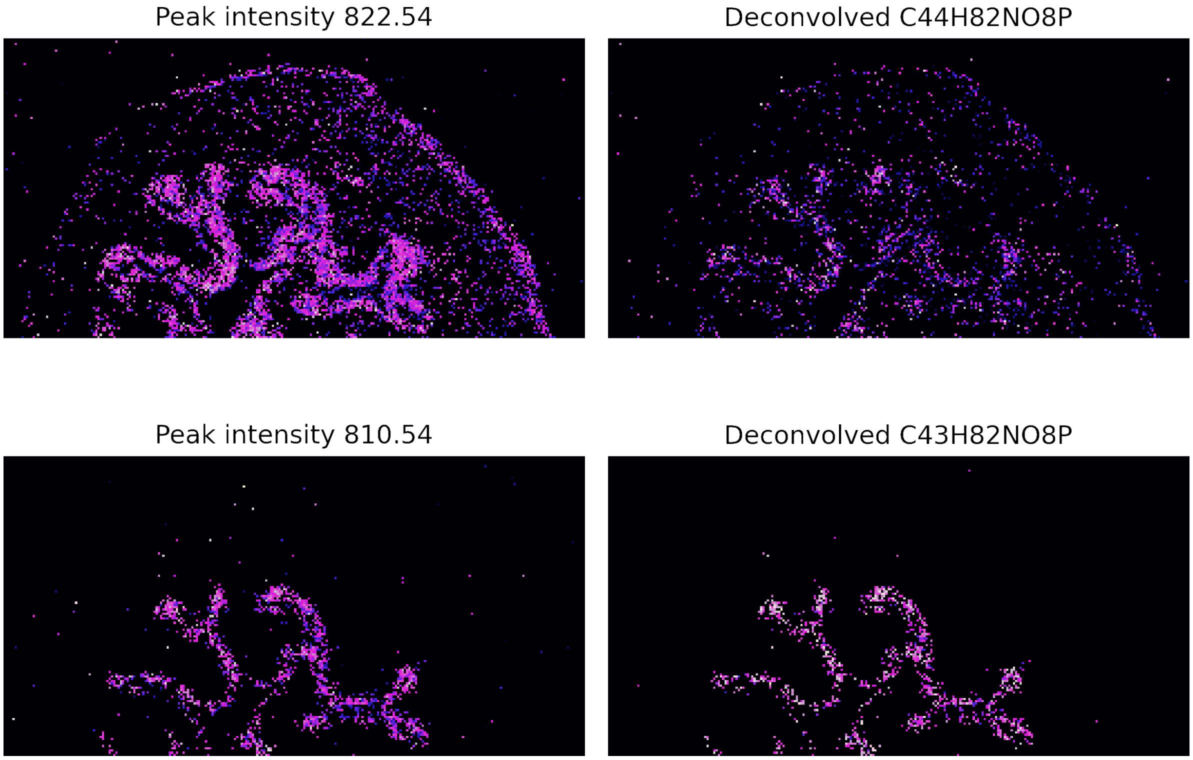

Supplementary Figure S12: Examples of ion images distorted due to overlapping isotopic envelope interference in the mouse bladder MSI dataset. The peak intensity of 822.54 Da suggests the analyte is distributed throughout the urothelium, but linear deconvolution with `masserstein` detected a lipid ion with a chemical formula C44H82NO8P (tentative PC(36:3)) localized mostly in the umbrella cells. The peak intensity of 810.54 Da is localized in the umbrella cells; the linear deconvolution with `masserstein` shows more details in the spatial distribution of the corresponding lipid with a molecular formula C43H82NO8P (tentative PC(35:2)), and reveals that the lipid is not distributed evenly with this tissue.

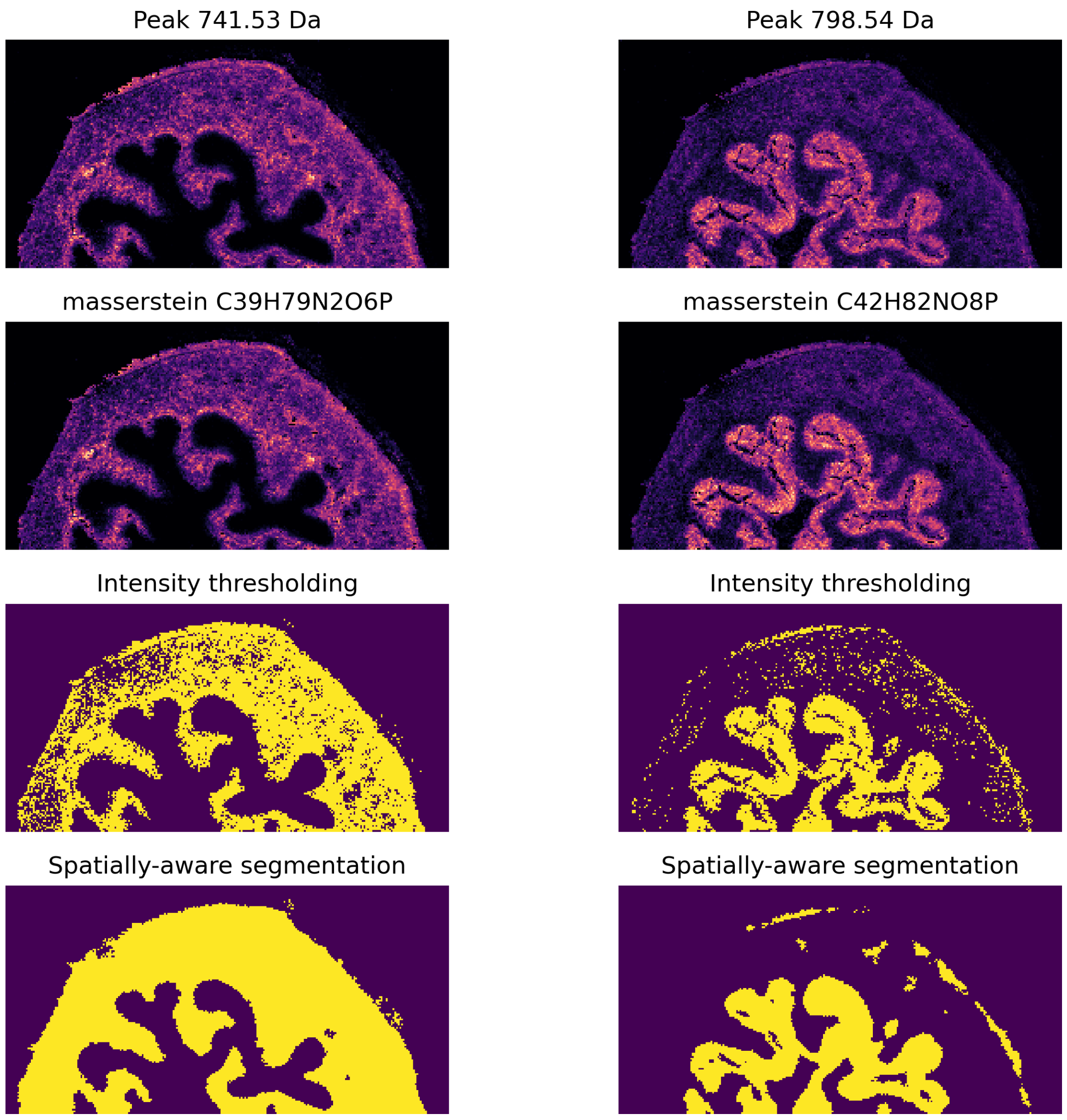

Supplementary Figure S13: Two examples of the impact of pixel-to-pixel variability on the spatially-naïve segmentation in the mouse bladder dataset (intensity thresholding with K-means), and the results after mitigating the variability with a spatially-aware segmentation algorithm `spatialDGMM`.

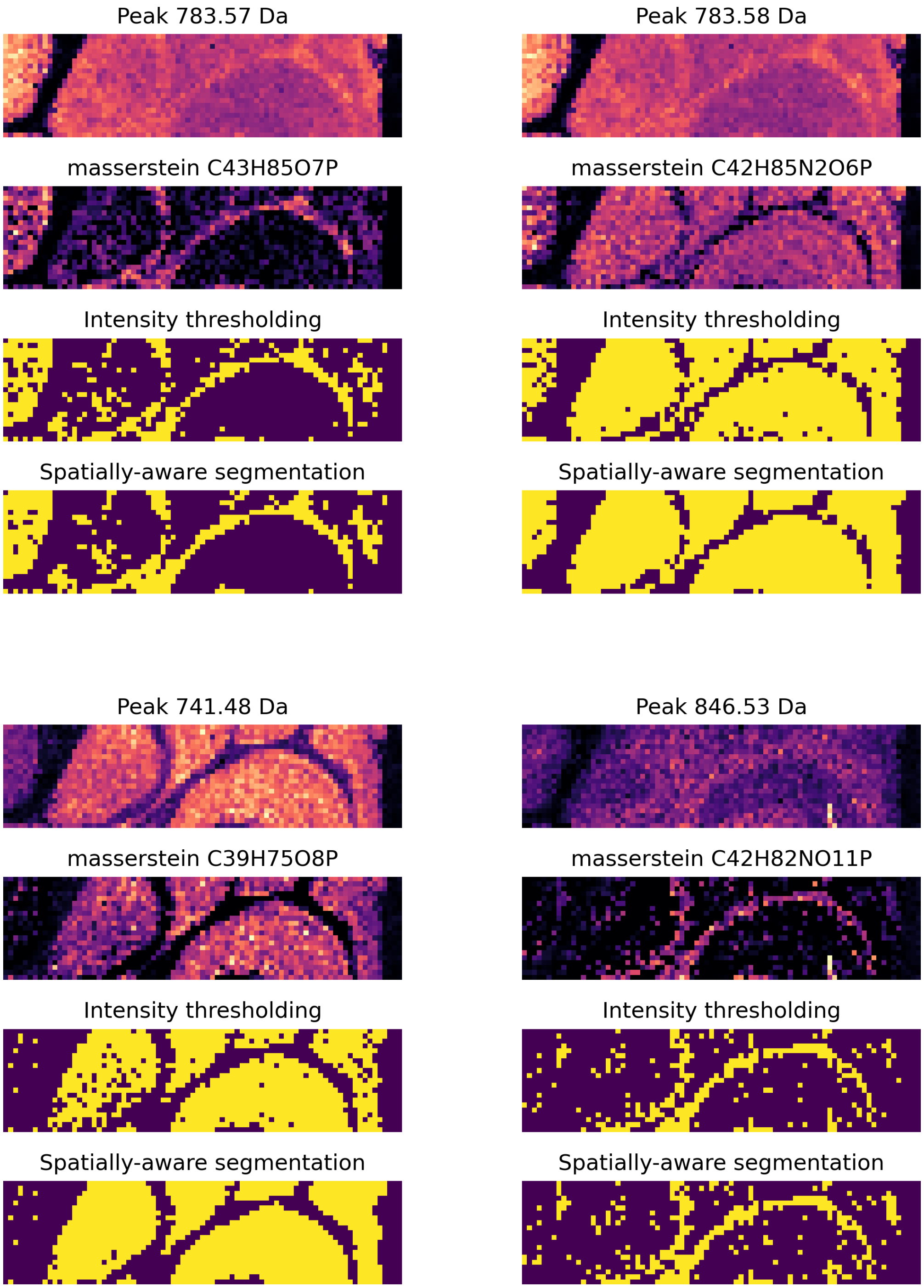

Supplementary Figure S14: Four examples of the impact of pixel-to-pixel variability and OIEs on a spatially-naïve segmentation of peak intensities in the mouse cerebellum dataset (peak intensity thresholding with K-means), and the results after deconvolving the ion images and mitigating the variability with a spatially-aware segmentation algorithm `spatialDGMM`.

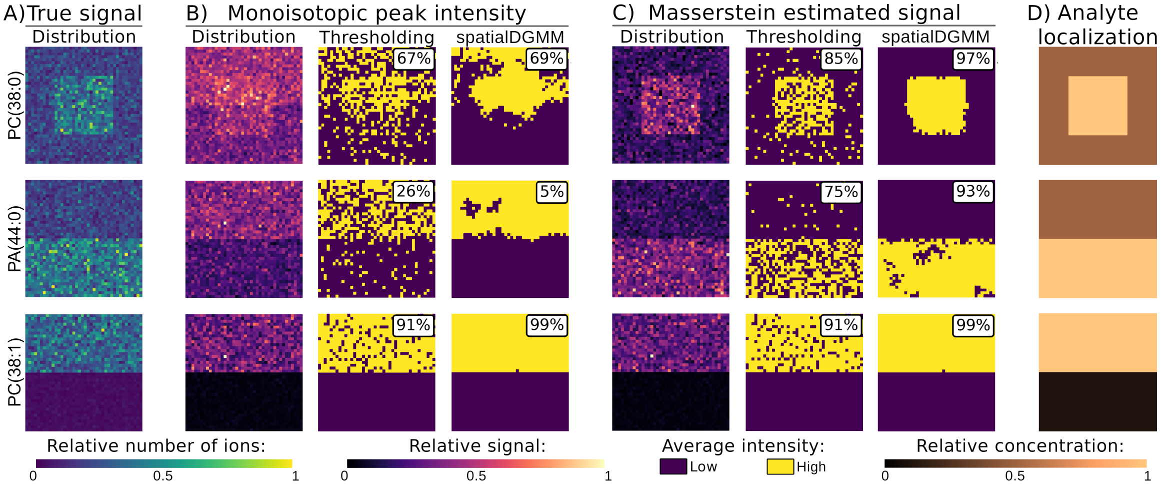

Supplementary Figure S15: From raw MSI data to the analyte localization regions. A) The simulated number of ions of each analyte. Each of the three lipids had a single region of concentration: a middle square for PC(38:0), bottom half for PA(44:0), top half for PC(38:1). B) Due to overlapping isotopic envelopes of the three lipids, the monoisotopic peak intensity of PA(44:0) was overshadowed by PC(38:1) and the one of PC(38:0) was distorted by the two other lipids. Consequently, segmentation through intensity thresholding suggested that PA(44:0) was concentrated in the top, rather than the bottom, half. Because the signal distribution is qualitatively incorrect, addressing the pixel-to-pixel variability further decreased the accuracy. C) Separating the overlapping isotopic envelopes with `masserstein` corrected the lipid spatial distributions. However, a spatially-naïve thresholding of the signals was insufficient to obtain an accurate segmentation. Using `spatialDGMM` to address the pixel-to-pixel variability of signals estimated with `masserstein` produced spatially homogeneous clusters with 99% of pixels correctly assigned to segments. The percentages of pixels correctly classified as low- or high-intensity are shown in the top-right corners of images. D) The true localization regions of the analytes used to simulate the numbers of ions in A).

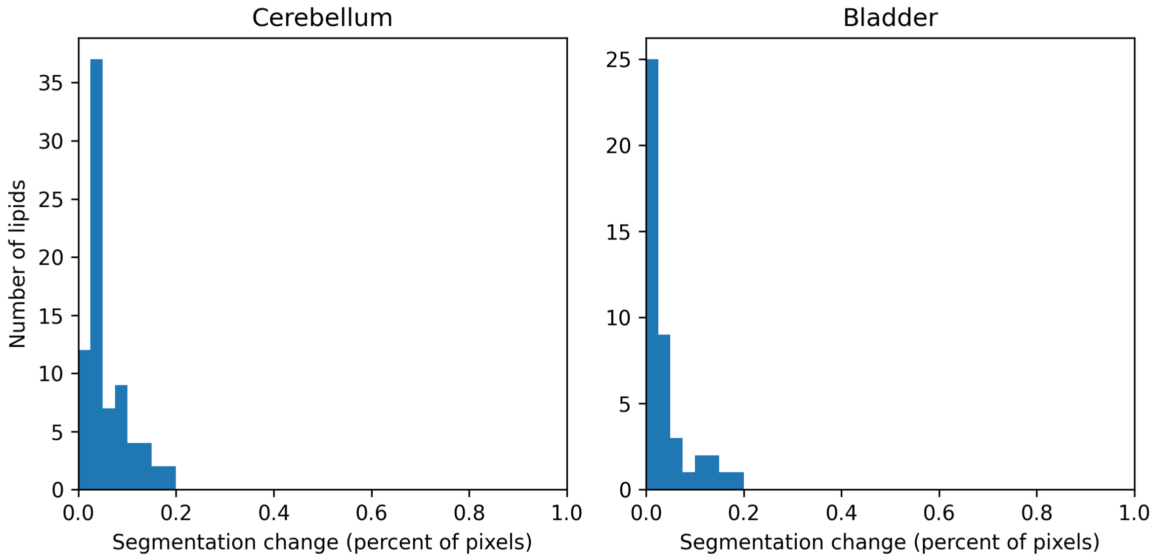

Supplementary Figure S16: The difference in segmentation between two univariate segmentation algorithms on the same input: the spatially-naïve K-means (which simply finds an intensity threshold to partition the dataset into low- and high-intensity segments), and the spatially-aware `spatialDGMM` (which assign posterior probabilities of segment assignment and smooths them using a spatial filter). Both algotithms were applied on the `masserstein`-estimated lipid proportions. The histograms show the percentage of pixels assigned to a different cluster (low- or high-intensity) by the segmentation algorithms.

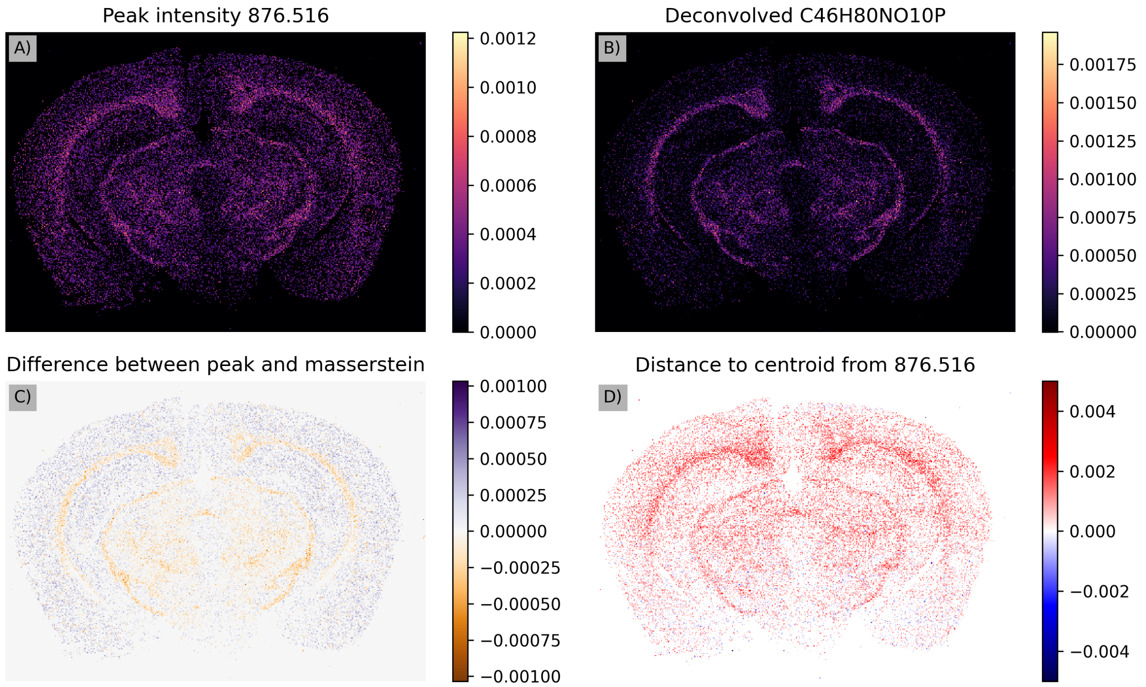

Supplementary Figure S17: A possible OIE interference in the Mouse Brain dataset for tentative PS(40:5). The single-ion image A) and the masserstein estimation B) indicate that the lipid ion is located in different tissues. The difference between the estimation methods C) coincides with anatomical features and with the distance between the monoisotopic mass of the lipid to the closest centroid in the pixel spectrum D). The correlation between anatomical features and the distance to the closest centroid D) indicates a systematic shift of the peak in the pixel spectra, suggesting the presence of an overlapping signal or isobaric interference from a different molecule.

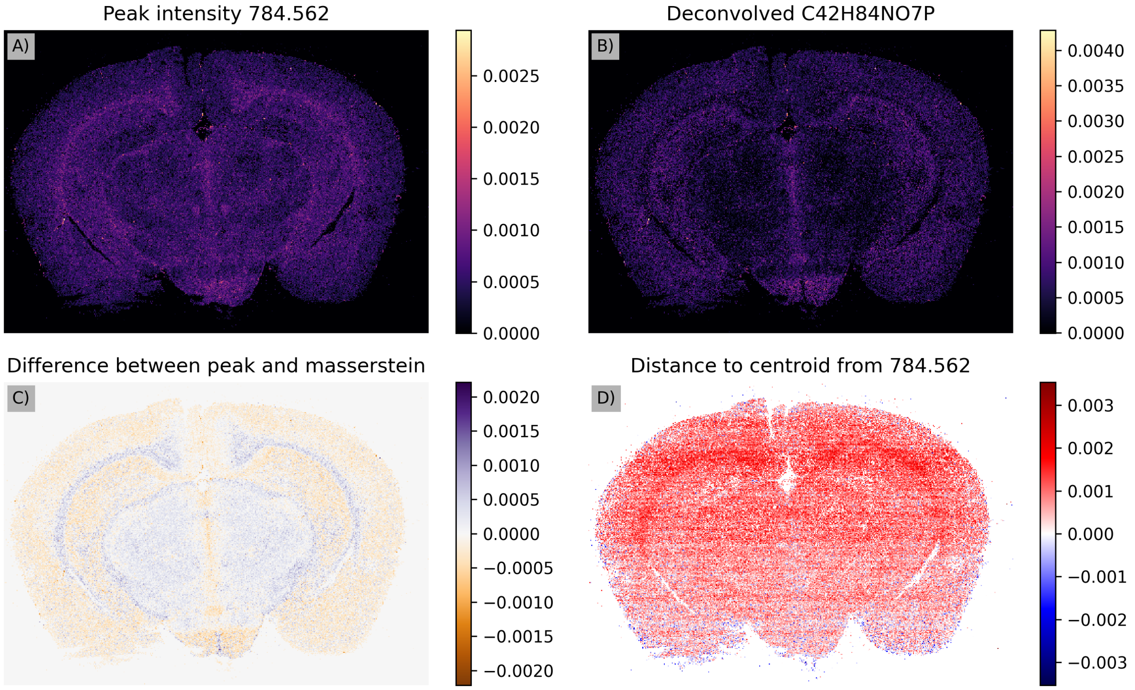

Supplementary Figure S18: A possible OIE interference in the Mouse Brain dataset for tentative PC(O-34:1). The single-ion image A) and the masserstein estimation B) indicate that the lipid ion is located in different tissues. The difference between the estimation methods C) coincides with anatomical features. Some correlation with the distance between the monoisotopic mass of the lipid to the closest centroid in the pixel spectrum D) is also visible. The correlation between anatomical features and the difference between the estimation methods, as well as systematic shift in the location of the closest centroid in some tissues, suggests interference from a different molecule.

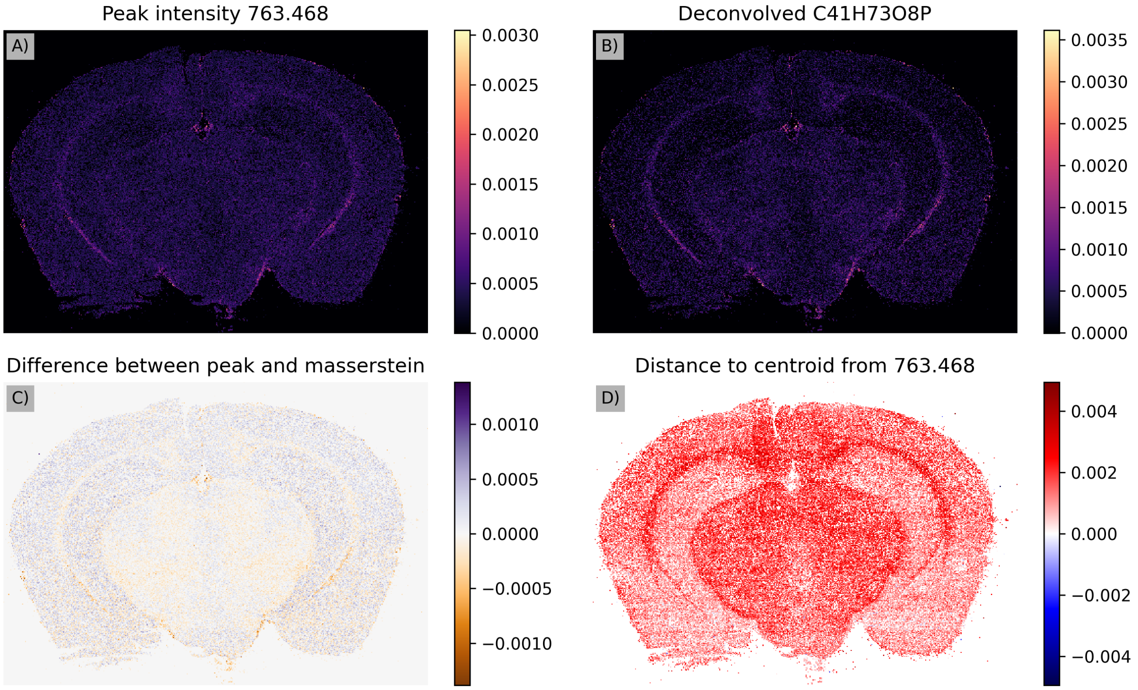

Supplementary Figure S19: A possible OIE interference in the Mouse Brain dataset for tentative PA(38:4). The single-ion image A) and the masserstein estimation B) show different concentrations of the ion in different tissues. The difference between the estimation methods C) coincides with anatomical features and with the distance between the monoisotopic mass of the lipid to the closest centroid in the pixel spectrum D). The correlation between anatomical features and the distance to the closest centroid D) indicates a systematic shift of the peak in the pixel spectra, suggesting the presence of an overlapping signal or isobaric interference from a different molecule.

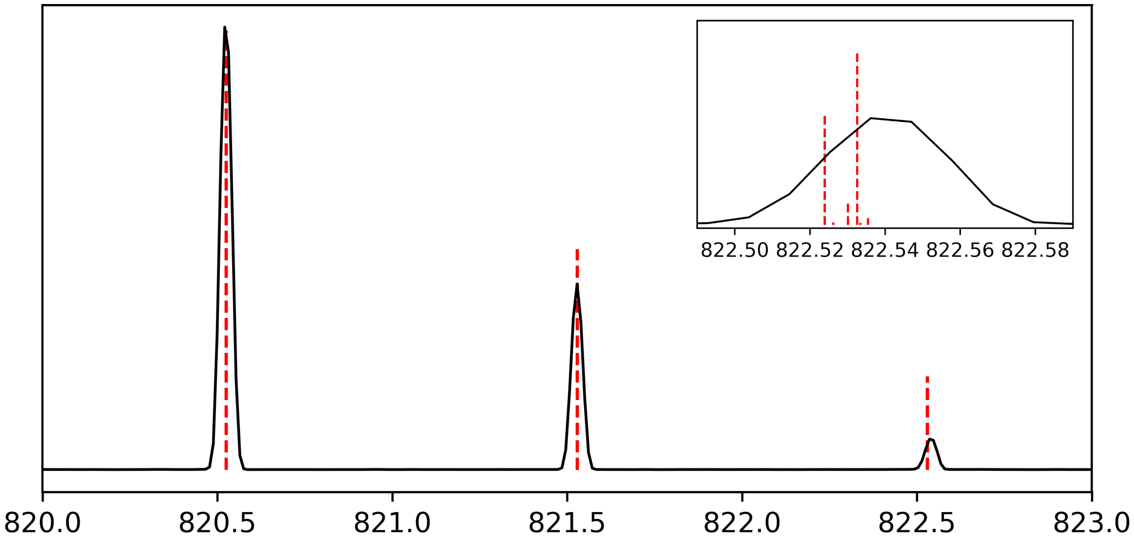

Supplementary Figure S20: In the mouse bladder MSI dataset, the average spectrum shows a disagreement with the theoretical isotope pattern of [PC(36:4)+K]. Although the first two peaks match almost exactly, the third theoretical peak is over two times too high and shifted to the left. Inspecting its fine isotopic distribution shows that the signal at 822.54 Da corresponds to multiple theoretical peaks, two of which form to the majority of the intensity. Out of these two major theoretical peaks, the lighter one corresponds to the presence of a ^{41}K isotope. However, the signal of this theoretical peak seems to be missing in the experimental spectrum and causes the apparent shift of intensity and location. This necessitates the use of linear deconvolution methods which are robust to these kinds of disagreements between experiments and theory. We remark that this is unlikely to be an error of the annotation, as lipids are ionized with potassium adducts in this dataset.
